# Home-based exercise in postmenopausal and older women: effects on body composition, physical function, cardiometabolic health, and quality of life—a systematic review and meta-analysis

**DOI:** 10.3389/fpubh.2026.1925532

**Published:** 2026-10-29

**Authors:** Yuhao Liu, Shuaifei Gu, Shaokai Tang

**Affiliations:** 1 College of Sports and Art, Jiangxi University of Science and Technology, Ganzhou, Jiangxi, China; 2 College of Sports Science, Jishou University, Jishou, Hunan, China

**Keywords:** body composition, cardiometabolic health, health-related quality of life, home-based exercise, meta-analysis, physical function, postmenopausal and older women

## Abstract

**Background:**

Home-based exercise (HBE) has received increasing attention as a low-cost and accessible strategy, although the durability of its effects and of long-term participation remains uncertain. Postmenopausal women frequently experience unfavorable changes in body composition, muscle strength, physical function, and cardiometabolic health. This review evaluated the effects of HBE on these outcomes and on health-related quality of life (HRQoL) in postmenopausal and older women.

**Methods:**

PubMed, Web of Science, Cochrane Library, and Embase were searched from inception to March 2026. Randomized controlled trials of home-based exercise in postmenopausal and older women were included. The review followed the PRISMA 2020 statement, was retrospectively registered in INPLASY, and used the Cochrane Risk of Bias 2 tool; data were synthesized in R using the meta package.

**Results:**

Nineteen randomized controlled trials involving 1,128 postmenopausal and older women were included. Pooled results showed that home-based exercise significantly reduced body mass index (BMI) (MD = −0.57 kg/m^2^, 95% CI: −0.71 to −0.43; I^2^ = 0%) and significantly improved health-related quality of life (HRQoL) (SMD = 0.51, 95% CI: 0.08 to 0.94; I^2^ = 76%). Effects on the Timed Up-and-Go test (TUGT) (MD = −0.64 s, 95% CI: −1.57 to 0.30), the five-times sit-to-stand test (5 × STS) (MD = −1.92 s, 95% CI: −4.36 to 0.52), handgrip strength (MD = 1.43 kg, 95% CI: −1.02 to 3.88), the six-minute walk test (MD = 35.16 m, 95% CI: −59.96 to 130.29), body fat percentage (MD = −0.31%, 95% CI: −0.65 to 0.03), and triglycerides (MD = −8.96 mg/dL, 95% CI: −19.45 to 1.52) did not reach statistical significance.

**Conclusion:**

Home-based exercise may reduce BMI and improve HRQoL in postmenopausal and older women, although the certainty of this evidence was moderate and very low, respectively. Effects on physical function and cardiometabolic markers remain inconclusive, with prediction intervals crossing the null for the most heterogeneous outcomes. Given the small number of trials per outcome, further well-designed randomized controlled trials with standardized prescriptions, longer follow-up, and routine adherence and adverse-event monitoring are needed.

**Systematic review registration:**

https://inplasy.com/inplasy-2026-4-0094/, identifier INPLASY202640094.

## 1 Introduction

Menopause is a major biological transition in the female life course and is characterized by progressive ovarian senescence, the permanent cessation of menstruation, and a sustained decline in circulating estrogen. The World Health Organization emphasizes that menopause should be understood within a life-course framework rather than as a disease, because postmenopausal health is shaped by previous lifestyle, reproductive history, social environment, and access to health services (1). As population aging accelerates, more women live for many years after menopause, making health support during the postmenopausal stage an increasingly important public health issue. Recent studies further indicate that menopausal symptoms, self-management capacity, and adherence to healthy behaviors may influence women’s life satisfaction, long-term health maintenance, and quality of life (2–4). Menopause-related endocrine changes may also influence multiple physiological systems and may increase the risks of cardiovascular disease, diabetes, metabolic syndrome, osteoporosis, obesity, sarcopenia, and skeletal muscle dysfunction (5–11). Postmenopausal women may therefore experience increased abdominal adiposity, reduced muscle mass, impaired lower-limb function, elevated blood pressure, and decreased physical activity capacity (5, 6, 12). These changes may compromise daily functioning and functional independence and may increase long-term health burden (9, 12). Therefore, safe, accessible, low-cost, and sustainable intervention strategies are needed to promote health and preserve function in postmenopausal and older women.

Physical inactivity may further amplify menopause-related health risks. Regular exercise is widely recognized as an effective non-pharmacological strategy for improving physical function, body composition, cardiorespiratory fitness, bone health, psychological well-being, and health-related quality of life (HRQoL) in middle-aged and older women (13–20). Aerobic exercise may contribute to improved cardiorespiratory capacity and fat control, whereas resistance training is particularly relevant for increasing muscle strength, maintaining lean mass, and supporting musculoskeletal health (16, 21, 22). Evidence also suggests that regular exercise is associated with a lower risk of osteoporosis in postmenopausal women and that physical activity is closely related to functional independence and HRQoL in older adults (11, 19, 20). Current physical activity guidelines recommend that adults and older adults accumulate regular aerobic physical activity and perform muscle-strengthening activities involving major muscle groups (23). For postmenopausal women, regular physical activity is therefore not only a disease-prevention strategy but also a practical approach to maintaining physical function, independence, and quality of life.

However, much of the existing evidence on exercise interventions has been generated from supervised, facility-based programs. Such programs may improve exercise safety, adherence, and training quality, but their implementation often requires fixed facilities, transportation, equipment, professional guidance, and sustained face-to-face management (24, 25). These requirements may limit scalability and long-term participation, particularly for women who are older, have reduced physical function, live far from exercise facilities, or have chronic conditions. In addition, adherence to physical activity during menopause may be affected by age, education, family responsibilities, health literacy, socioeconomic status, motivation, and perceived self-efficacy (2, 4). Remote physical activity support and online exercise management have been proposed as practical strategies to overcome access barriers, but their implementation remains influenced by digital literacy, technology acceptance, safety concerns, and the availability of appropriate guidance (26, 27). Therefore, relying solely on traditional supervised exercise models may be insufficient to meet the long-term health management needs of postmenopausal women.

Home-based exercise (HBE) is a structured form of exercise delivered primarily in the home environment, usually with minimal or no direct supervision. HBE may include aerobic training, resistance exercises, balance training, flexibility exercises, or multicomponent programs. Home-based and supervised programs appear to produce broadly comparable gains in older adults, although supervised delivery may be marginally superior for pain and disability (24). Compared with facility-based exercise, HBE requires fewer environmental and equipment resources and can be more easily integrated into daily routines. This delivery model may lower the threshold for participation, reduce travel and financial burden, and provide a more flexible option for postmenopausal women who have difficulty attending long-term supervised programs. With the development of telehealth, video-based instruction, mobile applications and home monitoring tools, HBE has gained increasing relevance in chronic disease management and health promotion; in community-dwelling older adults, digitally delivered home-based exercise has improved lower-limb strength, functional capacity and HRQoL and reduced falls (25). Recent evidence indicates that remotely supervised, videoconference-delivered exercise is feasible for older adults, with attendance of about 80%, and may improve physical function, muscle strength and quality of life (28), while also highlighting the need to consider technology-related barriers and individual preferences (26, 27, 29). Prior trials also suggest that HBE or remotely supported exercise may improve muscle strength, functional mobility, walking capacity, psychological well-being, and selected metabolic indicators in older or postmenopausal women (30, 31). Nevertheless, individual studies vary substantially in exercise modality, frequency, duration, supervision format, participant health status, adherence monitoring, and outcome measures, making it difficult to draw stable conclusions from single trials alone.

Several systematic reviews and meta-analyses have examined exercise interventions in postmenopausal women, but most have focused on specific exercise modalities or single outcome domains, such as body composition, bone mineral density, muscle strength, mind–body exercise, or cardiorespiratory fitness (13–16, 18). Two recent syntheses in postmenopausal women reached broadly similar conclusions: exercise improved menopausal symptoms and quality of life (32), but pooled effects on body mass index (BMI), blood pressure and measured physical performance were not statistically significant (33). In addition, some previous syntheses did not clearly distinguish home-based delivery from facility-based or supervised delivery (32, 33). The overall effects of HBE on multidimensional health outcomes in postmenopausal and older women therefore remain uncertain. This distinction is important because the public health value of an exercise intervention depends not only on its physiological efficacy but also on its accessibility, adherence, sustainability, and scalability. Existing evidence suggests that menopausal self-management, healthy lifestyle adherence, remote exercise support, and online exercise management are all relevant to long-term health promotion, but these studies do not directly quantify the pooled effects of HBE across body composition, physical function, cardiometabolic health, and HRQoL (3, 4, 26, 27, 29). A key question therefore remains unresolved: can HBE, with limited reliance on professional supervision and fixed facilities, improve multidimensional health outcomes in postmenopausal and older women?

Accordingly, this systematic review and meta-analysis aimed to evaluate the effects of HBE on body composition, physical function, cardiometabolic health, and HRQoL in postmenopausal and older women. We included randomized controlled trials and further explored whether intervention duration, training frequency, and participant health status might explain between-study heterogeneity. By synthesizing the available evidence, this study provides a more comprehensive basis for designing home-based exercise prescriptions, supporting long-term health management, and informing public health practice for postmenopausal and older women.

## 2 Methods

### 2.1 Information sources and search strategy

This systematic review and meta-analysis was reported in accordance with the Preferred Reporting Items for Systematic Reviews and Meta-Analyses (PRISMA) 2020 statement (34). The review was retrospectively registered with the International Platform of Registered Systematic Review and Meta-analysis Protocols (INPLASY; registration number: INPLASY202640094) on 26 April 2026, by which time the literature search and the quantitative analysis were largely complete. Deviations from the protocol are reported in the Supplementary material. PubMed, Web of Science, Cochrane Library, and Embase were systematically searched from database inception to March 2026. The search was rerun in September 2026 and identified two additional eligible trials (35, 36). The reference lists of the included trials and of relevant reviews were also screened for additional eligible studies.

The search strategy was developed by combining terms related to postmenopausal status, home-based delivery, exercise, and, where applicable, randomized or controlled trial design. The main search concepts included postmenopausal terms such as “Postmenopause,” “post-menopausal period,” “post menopause,” “older women,” and “menopausal women”; home-based delivery terms such as “home-based,” “in-home,” and “home”; and exercise-related terms such as “Exercise,” “physical exercise,” “aerobic exercise,” “exercise training,” and “physical activity.” Trial-related terms, including “randomized controlled trial,” “randomized,” “randomly assigned,” “controlled trial,” “clinical trial,” and “RCT,” were applied where appropriate. Database-specific controlled vocabulary, subject headings, text words, and field tags were adapted for each database. The reference lists of included studies and relevant reviews were also manually screened to identify additional eligible studies. The complete database-specific search strategies are provided in Supplementary Table S1. The menopausal concept included the free-text term “older women” in all four databases (Supplementary Table S1). This term was nested within the menopausal concept rather than developed as a separate older-adult concept, so the search was not designed to identify older-women trials indexed only under other age-related terms, such as “aged” or “community-dwelling older adults. Grade C trials are therefore reported as a *post hoc* extension of the registered population, and the review makes no claim to be a complete synthesis of exercise trials in older women irrespective of menopausal status.

### 2.2 Eligibility criteria

Eligibility criteria were originally prespecified according to the Population, Intervention, Comparator, Outcome and Study design (PICOS) framework. During revision, the population criterion was broadened from postmenopausal women to postmenopausal and older women; this protocol deviation is reported in the Supplementary material. The core inclusion criteria are summarized in Table 1 and are described below.

**Table 1:** PICOS eligibility criteria.

PICOS item	Criteria
Population	Postmenopausal and older women (mean or minimum age ≥60 years where menopausal status was not documented), with or without comorbidities such as osteoporosis or osteopenia, breast cancer survivorship, type 2 diabetes, obesity or metabolic risk, cardiovascular disease, or frailty. Postmenopausal status was defined as amenorrhoea for ≥12 consecutive months, bilateral oophorectomy, or STRAW+10 staging. The registered criterion was postmenopausal women; trials that enrolled older women without a documented menopause criterion were additionally retained as a protocol deviation, and are reported as grade C in Table 2.
Intervention	Structured home-based exercise (HBE) programs lasting ≥4 weeks, comprising aerobic, resistance, balance, flexibility or multicomponent training. At least 50% of prescribed sessions had to take place in the home environment; sessions delivered synchronously by video were counted as home-based. Trials in which a supervisor was present at ≥50% of sessions were excluded.
Comparator	Inactive control (usual care, no exercise, wait-list, or routine daily activity); attention control (identical investigator contact in both arms); or active exercise control (reported separately, not pooled).
Outcomes	Body composition (BMI, body fat percentage); health-related quality of life; physical function (TUGT, 5 × STS, handgrip strength, 6MWT); and cardiometabolic markers (triglycerides, SBP, DBP). Individual estimates for 6MWT, SBP and DBP are reported in Supplementary Table S2.
Timing	Outcomes were extracted at the end of the intervention period; where multiple follow-up time points were reported, the assessment immediately after the intervention was used preferentially. No minimum follow-up duration was required.
Study design	Randomized controlled trials.

Eligible participants were postmenopausal women. In addition to this registered criterion, and as a protocol deviation, trials that enrolled older women without any stated menopause criterion were retained and classified as grade C; the registered protocol specified postmenopausal women only and stated that women whose menopausal status was not documented would be excluded. Postmenopausal status was defined as amenorrhoea for at least 12 consecutive months, bilateral oophorectomy, or Staging of Reproductive Aging Workshop +10 (STRAW+10) staging (37). Each included trial was classified according to how menopausal status was established: grade A, an operational definition reported; grade B, described as postmenopausal without an ascertainment procedure; grade B*, no ascertainment procedure but all participants in an estrogen-deprivation treatment context; or grade C, older women only, with no menopause criterion stated. This classification is reported in Table 2. Eligible interventions were structured home-based exercise programs lasting at least 4 weeks. Home-based exercise was defined operationally as a program in which at least 50% of prescribed sessions are performed independently in the participant’s residence or its immediate surroundings; sessions delivered synchronously by video link were counted as home-based. Supervision was graded from L1 (written or recorded guidance only) to L4 (supervisor present for at least 50% of sessions); trials meeting the L4 criterion were excluded. Multi-arm trials contributed only their home-based arm. Exercise modalities could include aerobic training, resistance training, balance training, flexibility training, or multicomponent exercise.

**Table 2:** Study design and population.

Study	Country	Participants and age (*n*; years)	Population/comorbidity	Menopausal status ascertainment	Concomitant medication	Comparator	Comparator type
Tan et al. (58)	United Kingdom	10/10 (of 30, 3-arm); 57.7 ± 4.8/54.7 ± 3.3	Overweight/obesity (BMI > 25), otherwise healthy	A — ≥ 12 consecutive months amenorrhoea (self-reported)	NR	Daily activities	I
Çergel et al. (42)	Turkey	20/20; 60.20 ± 7.57/59.65 ± 6.45	Osteoporosis with vertebral fractures	A — postmenopausal ≥1 year	Osteoporosis drugs ≥6 months (all)	Daily activities	I
Chien et al. (49)	China	14/14; 61.7 ± 9.0/58.6 ± 9.3	Osteoporosis or osteopenia, without fracture	A — menopause ≥1 year	Calcium 600–900 mg/day (all); hormone therapy for 2-3 years (*n* = 2)	Daily activities	I
Gary and Lee (41)	United States	13/10; 68 ± 12/68 ± 12	Diastolic heart failure	C — no criterion; mean age 68 y	ACE inhibitors 61%, diuretics 83%, β-blockers 52%, CCB 39%; antidepressants 47%	Weekly home visits + education	II
Gibbs et al. (51)	Canada / Australia	71/70; 76.4 ± 6.4/77.0 ± 7.3	Osteoporosis (vertebral fracture)	C — no criterion; mean age 76–77 y	Vitamin D 1000 IU/day (all); osteoporosis drugs recorded	Usual care (equal attention)	II
Gorzelitz et al. (52)	United States	20/20; 60.9 ± 9.6/60.9 ± 8.0	Endometrial cancer survivor	C — no criterion; mean age 61 y	NR (not restricted or collected)	Wait list	I
Niemelä et al. (55)	Finland	26/25; 79.8 ± 3.4/80.7 ± 3.9	≈1 chronic condition (1.3 ± 0.9/1.0 ± 0.7)	C — no criterion; mean age 80–81 y	4.9 ± 3.3/4.5 ± 3.2 drugs (classes NR)	Usual care	I
Nishida et al. (63)	Japan	31/31; 70.4 ± 5.8/69.7 ± 6.6	Apparently healthy	B — described as postmenopausal, no procedure reported	Companion report (64): antihypertensives 4, lipid-lowering 2, oral antidiabetics 2, other 12	No exercise	I
Nyrop et al. (56)	United States	31/31; 63.3 ± 6.9/64.4 ± 9.7	Breast cancer survivor	B* — adjuvant aromatase inhibitor therapy	Aromatase inhibitor (all)	Daily activities	I
Jamali et al. (54)	Iran	46/39; 60.1 ± 6.2/61.0 ± 5.8	Type 2 diabetes mellitus	B — described as postmenopausal, no procedure reported	Glucose-lowering 95/100%, lipid-lowering 82/94%, antihypertensives 56/48%	Daily activities	I
Vestergaard et al. (60)	Denmark	25/28; 81.0 ± 3.3/82.7 ± 3.8	Multiple chronic conditions	C — no criterion; mean age 81–83 y	NR	Usual care	I
Kamide et al. (62)	Japan	23/27; 71.0 ± 3.8/70.9 ± 3.4	Healthy	C — no criterion; eligibility age ≥65 y	NR (prednisolone users excluded)	Usual care	I
Hongo et al. (53)	Japan	38/36; 67 ± 5/67 ± 7	Osteoporosis	B — described as postmenopausal, no procedure reported	NR (bisphosphonates/vitamin D excluded)	Daily activities	I
Dash et al. (50)	United States	69/71; 58.3 ± 4.7/58.4 ± 5.3	Obesity with metabolic syndrome	B — described as postmenopausal, no procedure reported	NR	Wait list	I
Solak et al. (65)	Turkey	24/24; 58.7 ± 7.1/55.6 ± 4.8	Healthy (postmenopausal)	B — described as postmenopausal, no procedure reported	NR (drugs affecting balance excluded)	Active: PNF exercise	III
Ahmadi and Jalilvand (61)	Iran	15/15; 65–75 (range)	Healthy	C — no criterion; age range 65–75 y	NR	Daily activities	I
Souza Filho et al. (57)	Brazil	38/37; 66.8 ± 3.5/66.6 ± 3.8	Stage I–II breast cancer (on hormone therapy)	B* — hormone therapy for breast cancer	Hormone therapy (all)	Monthly education lectures	II
Kurç et al. (36)	Turkey	20/20 (of 22/22 randomized); 54.7 ± 3.1/54.2 ± 4.6	Healthy early postmenopausal women	A — natural menopause within the preceding 5 y (amenorrhoea ≥12 mo)	NR	Education only	I
Kocamer et al. (35)	Turkey	15/15 (of 45 randomized); 58.7 ± 3.7/55.1 ± 3.8	Postmenopausal obesity (BMI 30–40 kg/m^2^)	A — no spontaneous menstruation ≥12 mo (natural or surgical)	NR	Education only	I

Participants and age are given as intervention/control (*n*; mean ± SD, years). Menopausal status: A, an operational definition was reported; B, described as postmenopausal without an ascertainment procedure; B*, an estrogen-deprivation treatment context; C, the older-women branch, with no menopause criterion and a mean or minimum age ≥60 years. Comparator: I, inactive; II, attention control (identical investigator contact in both arms); III, active exercise control, not entered into any pooled analysis. Supervision: L1, written or recorded guidance only; L2, regular remote contact; L3, intermittent on-site attendance; L4, a supervisor present at ≥50% of sessions. Nishida et al. (63) is a self-declared ancillary report of Ohta et al. (64), whereas Jamali et al. (54) and Vahed et al. (59) are treated as a single trial pending author confirmation; overlapping cohorts were counted once in all analyses. BFP, body fat percentage; CCB, calcium channel blocker; HRmax, maximal heart rate; HR reserve, heart-rate reserve; MET, metabolic equivalent; NR, not reported; PNF, proprioceptive neuromuscular facilitation; RM, repetition maximum; RPE, rating of perceived exertion.

Eligible comparator conditions included no exercise, usual care, routine daily activity, waiting-list control, or other comparator conditions reported in the original trials. Comparators were classified into three tiers: tier I, inactive (no exercise, usual care, wait-list, or education only); tier II, attention control (no exercise but comparable attention or contact); and tier III, active control (another exercise intervention). The main analysis pooled tiers I and II only; the single trial with an active comparator was not pooled and is described separately. The main outcome domains included HRQoL, body composition, physical function, and cardiometabolic health.

Studies were excluded if they were duplicate publications, conference abstracts, protocols without available outcome data, narrative reviews, single-arm studies, studies conducted in institutionalized populations, or studies in which the intervention was mainly facility-based with only a minor home component. Studies with insufficient data that could not be extracted from the full text or reasonably converted were excluded from the quantitative synthesis.

### 2.3 Study selection

All retrieved records were imported into EndNote X9 for duplicate removal. Two reviewers (SG and YL) independently screened titles and abstracts according to the prespecified inclusion and exclusion criteria to determine whether each record was eligible for full-text assessment. During the full-text review stage, disagreements were resolved through discussion. If consensus could not be reached, a senior reviewer (ST) was consulted for final judgment. The study selection process is presented in Figure 1.

**Figure 1:**
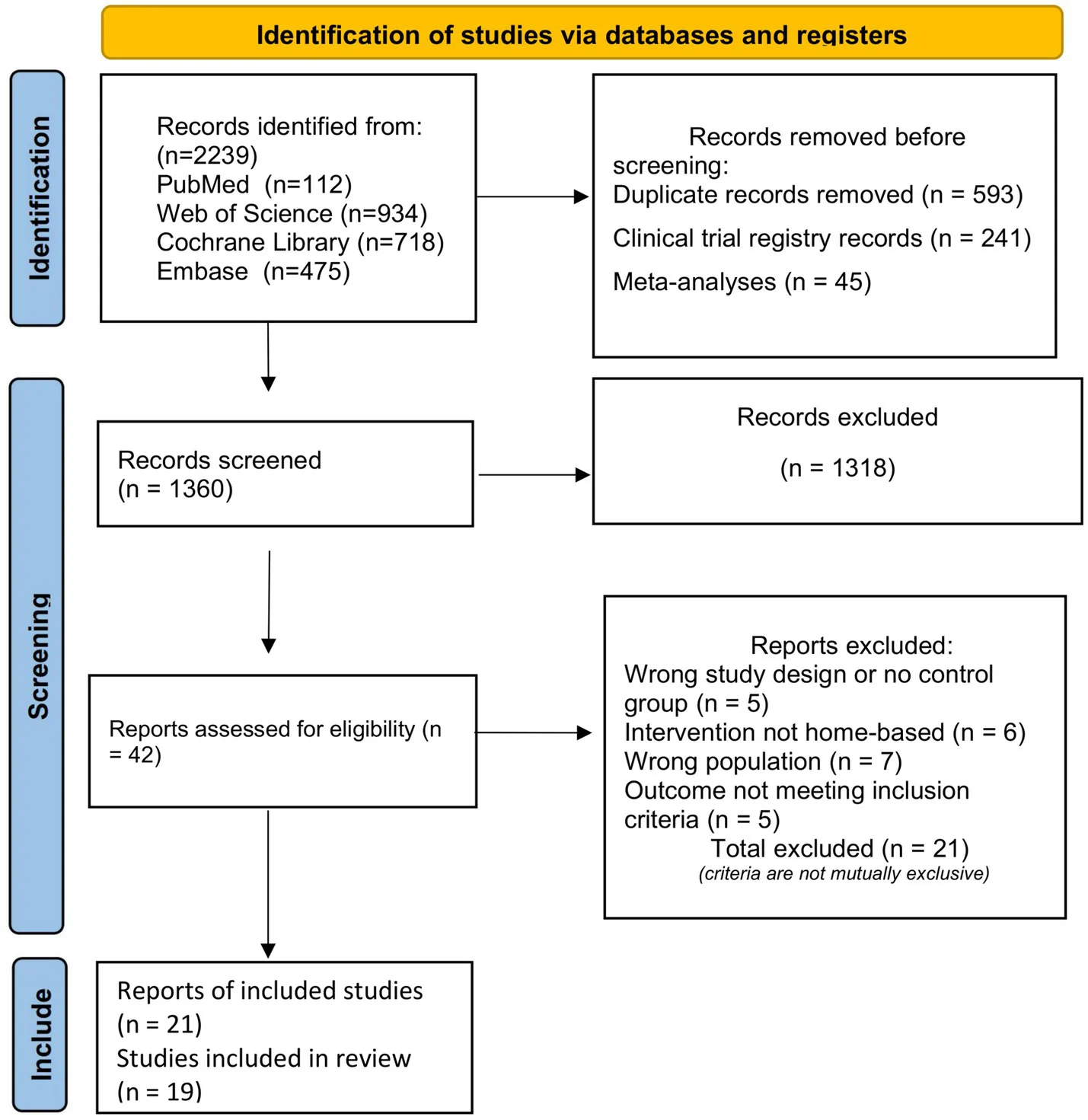
PRISMA 2020 flow diagram of study selection. The diagram shows the process of study identification, screening, eligibility assessment, and final inclusion in this systematic review and meta-analysis. Two pairs of articles reported the same underlying trials and were therefore counted once at the study level.

### 2.4 Data extraction

Two reviewers (SG and YL) independently extracted data using a predesigned standardized data extraction form. The extracted information included study characteristics, participant characteristics, intervention characteristics, comparator conditions, and outcome data. Specifically, study characteristics included the first author, publication year, country, and study design. Participant characteristics included the sample size in each group, mean age, standard deviation, and comorbidity profile. Intervention characteristics included the HBE modality, training frequency, exercise intensity, session duration, and total intervention duration. Comparator conditions included usual care, routine daily activity, no exercise intervention, or other eligible control conditions. Outcome data included the assessment time point and, for each group, the mean change from baseline and its standard deviation. When multiple follow-up time points were reported, the time point measured immediately after the intervention was extracted preferentially. Where a trial reported the change from baseline in each group together with its standard deviation, those values were used directly; where only the pre- and post-intervention means and standard deviations were available, the standard deviation of the change was derived from them assuming a within-participant correlation of 0.50, following methods recommended in the Cochrane Handbook (38). All pooled estimates therefore represent differences between groups in change from baseline. Any disagreement between the two reviewers during data extraction was resolved by rechecking the original article and discussion.

### 2.5 Risk-of-bias assessment

Two reviewers (SG and YL) independently assessed the risk of bias of the included studies using the Cochrane Risk of Bias 2 (RoB 2) tool (39). Five domains were evaluated: bias arising from the randomization process, deviations from intended interventions, missing outcome data, measurement of the outcome, and selection of the reported result. Each domain was judged as “low risk,” “some concerns,” or “high risk.” Any disagreement between the two reviewers was resolved by rechecking the original article and discussion. If consensus could not be reached, a third reviewer (ST) was consulted for final judgment. Risk-of-bias judgments are reported by domain; the RoB 2 tool provides a judgment about risk of bias rather than a grade of methodological quality.

### 2.6 Data synthesis and statistical analysis

As registered, the primary outcomes were HRQoL, BMI, handgrip strength, the Timed Up-and-Go test (TUGT) time and the five-times sit-to-stand test (5 × STS) time, and the secondary outcomes were body fat percentage, systolic blood pressure (SBP), diastolic blood pressure (DBP), serum triglycerides and six-minute walk test (6MWT) distance. All statistical analyses were performed using R software version 4.6 with the meta package (40). For outcomes measured using the same units across trials, including BMI, TUGT, 5 × STS, handgrip strength, body fat percentage, triglycerides, SBP, DBP and 6MWT, effect sizes were expressed as mean differences (MDs) with 95% confidence intervals (CIs). All 6MWT data were converted to meters (×0.3048 where the original unit was feet) and are reported in meters. For HRQoL, which was assessed with different instruments and scales, standardized mean differences (SMDs, Hedges’ g) with 95% CIs were calculated. Scores on the three instruments for which lower values indicate better HRQoL − the MLHFQ (41), the QUALEFFO-41 (42) and the MENQOL (35) − were multiplied by −1 so that higher values consistently indicate better HRQoL.

Between-study heterogeneity was assessed using the I^2^ statistic and Cochran’s Q test (43). A random-effects model was used for all outcomes; the choice of model was not based on the observed I^2^ or on the significance of the Q test. τ^2^ was estimated using restricted maximum likelihood (REML), and the Hartung–Knapp adjustment (44) was applied to the random-effects confidence interval because most outcomes were informed by few studies. τ^2^, τ, I^2^ and Cochran’s Q are reported for every outcome. Prediction intervals (45), based on the t-distribution with k − 1 degrees of freedom, are reported for outcomes with substantial heterogeneity. A fixed-effect model was fitted for methodological comparison only and was not used for any inference. Because the Hartung–Knapp variance is known to be anti-conservative when τ^2^ approaches zero and k is small (46), the Hartung–Knapp confidence intervals reported here are, if anything, slightly liberal for the outcomes with little heterogeneity.

Sensitivity analyses were conducted using the leave-one-out approach to examine the robustness of the pooled estimates. Prespecified sensitivity analyses also examined the restriction of the analysis to trials with documented menopausal status (grades A and B), the exclusion of all grade C trials, and the restriction to inactive comparators. For HRQoL, additional sensitivity analyses separated generic from disease-specific instruments, excluded trials reporting only a single SF-36 domain, and excluded trials judged to raise some concerns for outcome measurement. A further sensitivity analysis, added during revision at a reviewer’s request, excluded the two trials judged to be at high risk of bias overall; restriction of the analyses to the four trials judged to be at low risk of bias was not estimable for any outcome, because too few low-risk trials contributed to each. Exploratory subgroup analyses were performed according to comparator tier, menopausal status ascertainment, intervention duration, comorbidity, and session frequency; a formal test for subgroup differences was reported for each. A statistically significant between-subgroup difference does not by itself demonstrate effect modification (47), and subgroups containing fewer than two trials were not pooled. Because most outcomes included fewer than 10 studies, publication bias was assessed only for HRQoL; Egger’s regression test was performed and the result is reported with the caveat that its power is limited at this number of studies.

### 2.7 Certainty of evidence

The certainty of evidence for each outcome was assessed using the Grading of Recommendations Assessment, Development and Evaluation (GRADE) approach (48). Evidence from randomized controlled trials started at high certainty and was considered for downgrading across the five GRADE domains — risk of bias, inconsistency, indirectness, imprecision and publication bias — with certainty categorized as high, moderate, low or very low. Imprecision was assessed against an optimal information size derived from the pooled standard deviation and a minimal important difference for each outcome, as set out in Supplementary Table S3. The assessment was performed as part of the revision and was informed by the risk-of-bias assessment described in Section 2.5. The complete evidence profile, the input data and the reasons for each judgment are presented in Supplementary Table S3, and the resulting certainty ratings are summarized in Table 3.

**Table 3:** Summary of meta-analysis results.

Outcome	No. of trials	Participants	Effect estimate (95% CI)	Prediction interval	I^2^	p	Certainty (GRADE)
BMI (kg/m^2^)	4	197	MD − 0.57 (−0.71 to −0.43)	—	0%	<0.001	Moderate
Body fat percentage (%)	4	195	MD − 0.31 (−0.65 to 0.03)	—	0%	0.06	Low
HRQoL (SMD)	10	463	SMD 0.51 (0.08 to 0.94)	−0.75 to 1.78	76%	0.025	Very low
TUGT (s)	4	261	MD − 0.64 (−1.57 to 0.30)	−2.69 to 1.42	83%	0.12	Very low
5 × STS (s)	4	317	MD − 1.92 (−4.36 to 0.52)	—	43%	0.09	Low
Handgrip strength (kg)	4	226	MD 1.43 (−1.02 to 3.88)	—	43%	0.16	Low
6MWT (m)	3	133	MD 35.16 (−59.96 to 130.29)	−151.67 to 222.00	87%	0.25	Very low
Triglycerides (mg/dL)	3	232	MD − 8.96 (−19.45 to 1.52)	—	0%	0.07	Very low

All analyses used a random-effects model (REML estimator for τ^2^) with the Hartung–Knapp small-sample adjustment. Prediction intervals were calculated only for outcomes with substantial heterogeneity; “—” indicates that no prediction interval was calculated. BFP, body fat percentage; BMI, body mass index; HRQoL, health-related quality of life; MD, mean difference; SMD, standardized mean difference. SBP and DBP were each reported by only two trials and are presented individually in Supplementary Table S2.

**Table 4:** Home-based exercise prescription.

Study	Exercise mode	Intensity	Session duration (min)	Frequency	Program duration	Progression	Equipment	Supervision level
Tan et al. (58)	Equipment-free home HIIT (self-chosen or YouTube video)	High (≥80% HRmax; RPE ≥ 17; achieved 18)	20	3/wk (24 total)	8 wk	Yes — low-intensity intervals ↓ wks 1–4, maintained wks 5–8	None; Garmin Forerunner 35 (monitoring)	L2 — WhatsApp self-report + fortnightly meetings
Çergel et al. (42)	Home back-extensor strengthening (4 movements)	NR	NR	3/wk	6 wk	Yes — 3 × 8 → 3 × 10 → 3 × 12 reps	None; illustrated booklet + exercise chart	L1 — one-off demo + booklet; no follow-up
Chien et al. (49)	Home trunk strengthening (isometric abdominal/gluteal, bridging, prone extension)	NR (self-paced)	NR	3/day, 7 d/wk	12 wk	Yes — hold 3 → 10 s; reps and sets ↑	None; booklet + logbook	L2 — logbook + telephone every 2 wk
Gary and Lee (41)	Home progressive walking (warm-up, individualised endurance, cool-down)	40 → 50 → 60% HR reserve (Karvonen); RPE monitored	≥30 by wk 12 (goal)	3 d/wk	12 wk	Yes — + 10% once walking 20 min at prior intensity for 1 wk	Polar Beat HR watch; walking logs	L3 — weekly home visits by investigators
Gibbs et al. (51)	Multicomponent: upper/lower-body strength, posture/core, balance + daily aerobic	8–12 RM (start ≥2 sets × 8–10 RM)	NR (aerobic 10–30/day)	≥3/wk + daily aerobic	12 mo	Yes — progressed by physiotherapist at visits and calls	Resistance tubing	L3 — 6 physiotherapist visits + telephone calls
Gorzelitz et al. (52)	Home full-body resistance (8 exercises, all 5 muscle groups)	NR (load ∝ body size and strength)	NR	2/wk	10 wk	NR	Bands + dumbbells (5–12 lb); YouTube channel; printed manual	L1 — one in-person teaching visit
Niemelä et al. (55)	Home spring rocking-chair training (10 movements)	NR (self-selected, unmonitored)	15	10/wk (2/day × 5 d)	6 wk	Yes — speed and resistance ↑	Spring rocking-chair; written instructions; exercise diary	L2 — one telephone call (wk 3) only
Nishida et al. (63)	Home bench step exercise (15–20 cm) with a rhythm CD	Individual lactate threshold (4.18 ± 0.89/4.1 ± 0.66 METs)	10–20	3/day (target ≥140/wk)	12 wk	Yes — step test at wk 6, load readjusted	Bench step 15–20 cm; rhythm CD; exercise logs	L3 — weekly on-site group session
Nyrop et al. (56)	Self-directed home walking (Walk With Ease-Breast Cancer)	NR (not collected)	NR	≥150/wk	6 wk	NR	Workbook, brochure, printed activity log	L1 — no contact during intervention
Jamali et al. (54)	Home multi-task training (aerobic walking + dual task, resistance, balance, flexibility)	40–60% HR reserve; 13-point RPE scale	NR (30 walk)	3–5/wk (3 → 4 → 5)	12 wk (48 sessions)	Yes — resistance 1 × 8 → 3 × 10–12 reps; intensity 40–50 → 60% HR reserve	None; booklet + instructional videos	L2 — weekly telephone + training checklist
Vestergaard et al. (60)	Video-guided (15 min warm-up, 6 min elastic-band strength, 5 min aerobic)	NR (Borg discontinued)	26	3/wk (60 total)	5 mo	Minimal — more repetitions only	Elastic band; videotape; booklet; television/video player	L2 — instructor at first session; bi-weekly calls
Kamide et al. (62)	Home multicomponent (Thera-Band strength, balance stepping, heel drops)	Moderate; 1–2 sets × 15 reps; 60–100 heel drops	NR	≥3/wk	6 mo	No — content and intensity unchanged	Thera-Band; log sheets mailed monthly	L2 — monthly telephone/mail contact
Hongo et al. (53)	Home back-extensor training (prone trunk extension, 5-s holds)	Low; no additional weight	3–5	1 set/day, 5 d/wk	4 mo	NR (fixed single movement)	Bed + pillow; no loading equipment	L2 — telephone every 2 wk
Dash et al. (50)	Home walking (brisk walking or slow jogging) to a daily step goal	RPE 11–14 (20-point scale)	NR (7,000–10,000 steps/day)	Daily (≥150/wk)	6 mo	NR (fixed target volume)	Pedometer (self-monitoring)	L1 — baseline teaching only; no contact
Solak et al. (65)	Home PNF balance/coordination (4 hip patterns) vs. Frenkel exercises	NR	30 (PNF arm); NR (Frenkel arm)	5/wk	4 wk	NR	PNF arm: manual resistance + audiovisual biofeedback; Frenkel arm: brochure	Mixed — PNF L4 (therapist present); Frenkel L1
Ahmadi and Jalilvand (61)	Smartphone-delivered home aerobic (warm-up 10, aerobic 30, cool-down 5)	NR (tempo 40 → 90 beats/min)	45	3/wk	8 wk	Yes — movements and reps ↑ weekly; simple → complex	None; delivered via WhatsApp/Telegram	L2 — videos sent; completion reported in group chat
Souza Filho et al. (57)	Self-directed program (manual + DVD): range of motion, muscular fitness, walking	NR	30	≥5/wk	12 wk	Yes — weekly progressive routine	Printed manual + DVD	L2 — telephone twice weekly
Kurç et al. (36)	App-guided aerobic + resistance + core stabilisation + balance	Moderate (RPE 11–13; 40–60% HR reserve)	Aerobic 15 → 30	3/wk	8 wk	Yes — 2 × 10 → 3 × 12 reps at wk 5	None (bodyweight; chair and wall)	L1 — app video/audio cues only
Kocamer et al. (35)	Home mat Pilates + TheraBand resistance	CR10 RPE + talk test; band light → low-moderate → moderate	60	2/wk	8 wk (16 sessions)	Yes — advanced every 2 wk	Mat + TheraBand; illustrated brochure	L2 — 1 of 2 weekly sessions video-supervised

Participants and age are given as intervention/control (*n*; mean ± SD, years). Menopausal status: A, an operational definition was reported; B, described as postmenopausal without an ascertainment procedure; B*, an estrogen-deprivation treatment context; C, the older-women branch, with no menopause criterion and a mean or minimum age ≥60 years. Comparator: I, inactive; II, attention control (identical investigator contact in both arms); III, active exercise control, not entered into any pooled analysis. Supervision: L1, written or recorded guidance only; L2, regular remote contact; L3, intermittent on-site attendance; L4, a supervisor present at ≥50% of sessions. Nishida et al. (63) is a self-declared ancillary report of Ohta et al. (64), whereas Jamali et al. (54) and Vahed et al. (59) are treated as a single trial pending author confirmation; overlapping cohorts were counted once in all analyses. BFP, body fat percentage; CCB, calcium channel blocker; HRmax, maximal heart rate; HR reserve, heart-rate reserve; MET, metabolic equivalent; NR, not reported; PNF, proprioceptive neuromuscular facilitation; RM, repetition maximum; RPE, rating of perceived exertion.

## 3 Results

### 3.1 Study selection

Figure 1 presents the PRISMA 2020 flow diagram, illustrating the detailed screening process. The database search identified 2,239 records. After removal of 593 duplicates, 241 trial registry records and 45 meta-analyses, 1,360 records remained for title and abstract screening. Of these, 1,318 were excluded at the title and abstract stage and 42 full-text articles were assessed for eligibility; 21 were then excluded (wrong study design or no control group, *n* = 5; intervention not home-based, *n* = 6; wrong population, *n* = 7; outcome not meeting the inclusion criteria, *n* = 5; these categories are not mutually exclusive, so the counts sum to more than the number of articles excluded). Nineteen randomized controlled trials, reported in 21 articles, were finally included. The included studies were published between 2005 and 2026.

### 3.2 Study characteristics

The included studies span a period of 21 years, with their characteristics summarized in Tables 2–5. These studies were conducted in 10 countries, including Japan, the United States, Iran, Turkey, Brazil, China, Denmark, Finland, Canada/Australia, and the United Kingdom, collectively involving 1,128 postmenopausal and older women. The key characteristics of the studies are as follows: (1) Population: all studies enrolled postmenopausal or older women; 14 trials enrolled participants with comorbidities, such as osteoporosis or osteopenia, breast cancer survivorship, type 2 diabetes, pre-frailty, obesity or metabolic risk, diastolic heart failure, endometrial cancer survivorship, or multiple chronic conditions (35, 41, 42, 49–60), whereas five trials enrolled healthy postmenopausal or older women (36, 61–65). These two lists contain 21 references for 19 trials because two trials are each reported in a pair of articles [Vahed et al. (59) with Jamali et al. (54); Ohta et al. (64) with Nishida et al. (63)]; each pair was counted once throughout. (2) Age: all studies reported participant age, with mean ages ranging from 54.2 to 82.7 years. (3) Interventions: the intervention approaches were primarily home-based exercise (HBE) programs, including aerobic training, resistance training, trunk-strengthening exercise, balance exercise, stretching, bench-step exercise, walking programs, and video- or internet-based exercise. (4) Training frequency: all studies reported exercise frequency, which ranged from two sessions per week to three sessions per day. (5) Intervention duration: all included studies reported the total intervention duration, ranging from 4 to 52 weeks, with a median duration of 12 weeks. (6) Outcome measures: the studies employed a broad set of health-related outcomes, including body mass index (BMI) (35, 54, 58, 59, 63, 64), body fat percentage (BFP) (35, 36, 52, 54, 59), Timed Up-and-Go test (TUGT) (42, 51, 61, 62), five-times sit-to-stand test (5 × STS) (51, 54, 55, 60), handgrip strength (52, 54, 60, 62), six-minute walk test (6MWT) (41, 52, 54, 59), triglycerides (35, 50, 63, 64), systolic blood pressure (SBP) and diastolic blood pressure (DBP) (54, 59, 63, 64), and health-related quality of life (HRQoL) (35, 36, 41, 42, 49, 52, 53, 56, 57, 60). Supervision was graded L1 in five trials, L2 in 10 trials and L3 in three trials; the remaining trial combined an L1 arm and an L4 arm and was excluded from the main analysis because its comparator was an active exercise intervention.

**Table 5:** Implementation, safety and outcomes.

Study	Home-based (%)	Adherence (%)	Adverse events	Cohort overlap	Outcomes in pooled analysis
Tan et al. (58)	100	85 (EX); 96 (EX+D)	None	None	BMI
Çergel et al. (42)	100	>85 (attendance); 60/60 completed	NR	None	TUGT, QUALEFFO-41 (HRQoL)
Chien et al. (49)	100	>90 (range 86.9–98.5); 78.6% completed	None	None	SF-36 (HRQoL)
Gary and Lee (41)	100	NR (“similar” between groups)	NR	None	MLHFQ (HRQoL)
Gibbs et al. (51)	100	66 (per-protocol); 73 (per-diary)	NR (reported elsewhere)	None	5 × STS, TUGT
Gorzelitz et al. (52)	100	96 (19.2/20 sessions); 75% did ≥2/wk	NR (companion paper)	Companion paper Gorzelitz et al. (52) reports the same trial	FACT-En (HRQoL), 6MWT, BFP, handgrip
Niemelä et al. (55)	100	96 (86% did ≥10/wk)	NR (collected but not reported)	None	5 × STS
Nishida et al. (63)	≈95	93% reached target (companion report: 84.6%)	NR	Same trial as Ohta et al. (64); self-declared ancillary report	BMI, SBP, DBP, triglycerides
Nyrop et al. (56)	100	Not reported; 77% completed; walking 32.5 → 108.7 min/wk	None	None	FACT-G (HRQoL)
Jamali et al. (54)	100	83%; 85% completed (companion report: 84 and 70%)	Mild — generalized soreness/bruising in the initial weeks	Same trial as Vahed et al. (59); author confirmation pending	BMI, 5 × STS, 6MWT, SBP, DBP, BFP, handgrip
Vestergaard et al. (60)	100	89.2 (SD 13.3); 83% completed	None	None	EQ-5D (HRQoL), 5 × STS, handgrip
Kamide et al. (62)	100	82.6 (≥3 d/wk); 91.3 (≥2 d/wk)	None during intervention; 0 vs. 1 fall (control) at 12 mo	None	TUGT, handgrip
Hongo et al. (53)	100	Not quantified (“high compliance”)	Serious — 1 fall → forearm fracture; 3 transient back pain; no new vertebral fracture	None	JOQOL (HRQoL)
Dash et al. (50)	100	68 (≥80% of target)	NR	None	Triglycerides
Solak et al. (65)	Frenkel 100; PNF 0	NR	None	None	Not pooled (active-comparator layer); SF-36, handgrip narratively
Ahmadi and Jalilvand (61)	100	NR	NR	None	TUGT
Souza Filho et al. (57)	100	81.5	Mild — muscle soreness, resolved in ≈1.5 wk	None	EORTC QLQ-C30 (HRQoL)
Kurç et al. (36)	100	Not retrievable (app records deleted)	None	None	SF-36 Mental Health (HRQoL), BFP
Kocamer et al. (35)	100	100	None	None	MENQOL (HRQoL), triglycerides, BMI, BFP

Participants and age are given as intervention/control (*n*; mean ± SD, years). Menopausal status: A, an operational definition was reported; B, described as postmenopausal without an ascertainment procedure; B*, an estrogen-deprivation treatment context; C, the older-women branch, with no menopause criterion and a mean or minimum age ≥60 years. Comparator: I, inactive; II, attention control (identical investigator contact in both arms); III, active exercise control, not entered into any pooled analysis. Supervision: L1, written or recorded guidance only; L2, regular remote contact; L3, intermittent on-site attendance; L4, a supervisor present at ≥50% of sessions. Nishida et al. (63) is a self-declared ancillary report of Ohta et al. (64), whereas Jamali et al. (54) and Vahed et al. (59) are treated as a single trial pending author confirmation; overlapping cohorts were counted once in all analyses. BFP, body fat percentage; CCB, calcium channel blocker; HRmax, maximal heart rate; HR reserve, heart-rate reserve; MET, metabolic equivalent; NR, not reported; PNF, proprioceptive neuromuscular facilitation; RM, repetition maximum; RPE, rating of perceived exertion.

In addition to these primary outcomes, several studies used disease-specific or function-specific instruments to assess multidimensional health status, including quality-of-life scales, functional mobility tests, lower-limb performance tests, walking capacity measures, body composition indicators, and cardiometabolic markers. These indicators provided a comprehensive framework for evaluating the effects of HBE on body composition, physical function, cardiometabolic health, and HRQoL in postmenopausal and older women.

### 3.3 Risk of bias

Risk of bias was assessed with the Cochrane RoB 2 tool for all 19 included trials. The judgments for individual trials are presented in Figure 2B, and the distribution of judgments across domains in Figure 2A.

**Figure 2:**
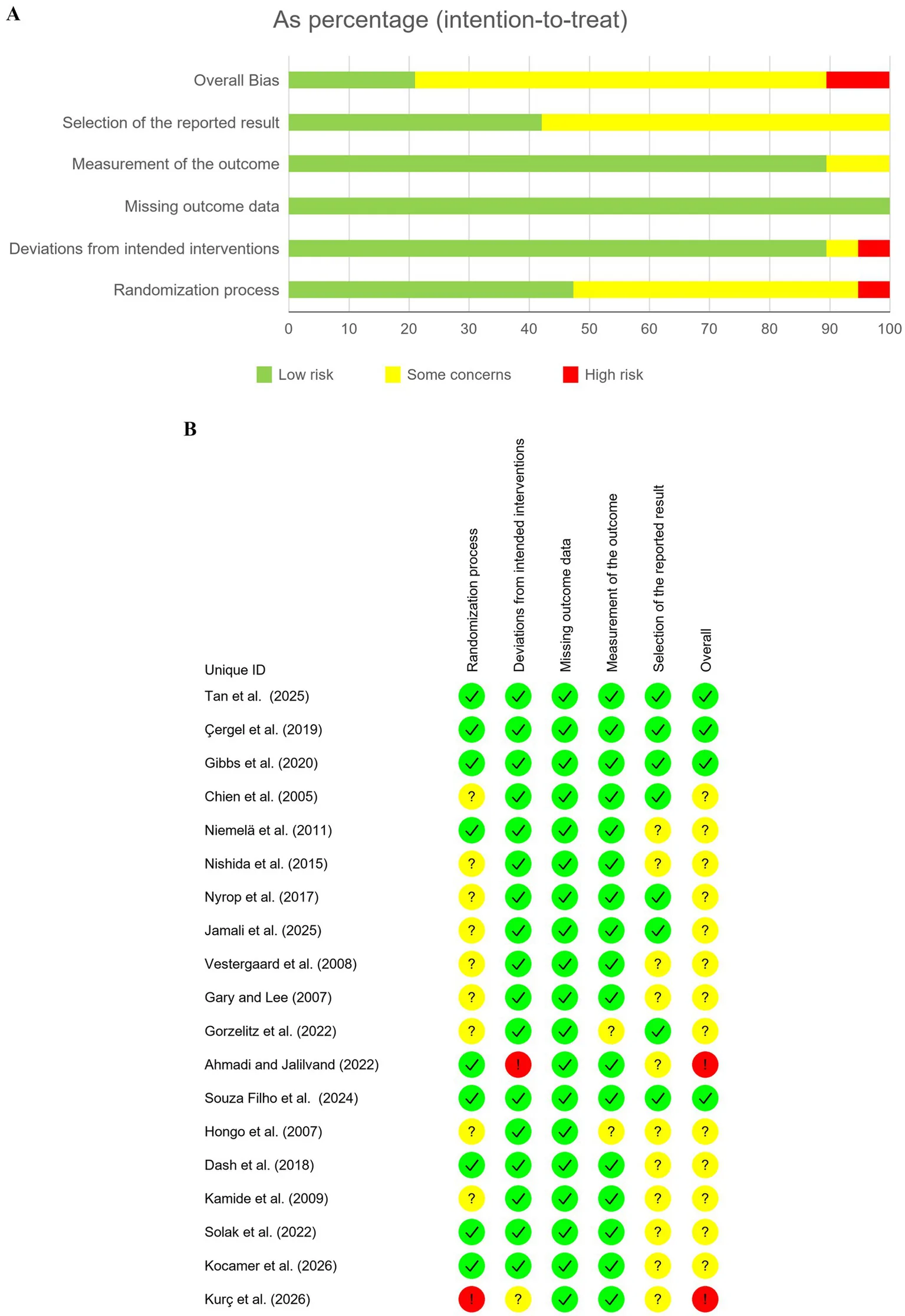
**(A)** Risk-of-bias bar plot. The figure summarizes the proportion of judgments across RoB 2 domains. **(B)** Risk-of-bias traffic-light plot. The figure shows domain-level risk-of-bias judgments for each included randomized controlled trial.

Overall, four trials (21.1%) were judged to be at low risk of bias (42, 51, 57, 58), 13 (68.4%) raised some concerns, and two (10.5%) were at high risk of bias (36, 61).

Selection of the reported result was the most frequent source of concern: 11 trials (57.9%) raised some concerns and eight (42.1%) were judged to be at low risk. In most of these trials no trial protocol or prespecified statistical analysis plan was available, so it could not be verified that the reported results corresponded to a prespecified analysis. No trial was judged to be at high risk in this domain.

Bias arising from the randomization process was the next most frequent concern, with nine trials (47.4%) raising some concerns and one trial (5.3%) judged to be at high risk. In most of these trials the method used to generate the random sequence, or the method used to conceal the allocation, was not reported in sufficient detail.

Deviations from the intended interventions rarely raised concern: 17 trials (89.5%) were judged to be at low risk, one trial (5.3%) raised some concerns, and one trial (5.3%) was at high risk. Although blinding of participants and personnel is not feasible in exercise trials, most trials reported an analysis consistent with the intention-to-treat principle, whereas no information was available in the trial judged to be at high risk on whether an appropriate analysis had been used to estimate the effect of assignment to intervention.

Missing outcome data raised no concern in any trial (19 of 19, 100%), as attrition was low or was accounted for in the analysis.

Measurement of the outcome raised some concerns in two trials (10.5%), in which outcome assessors were not blinded, or the outcome was self-reported without further safeguards.

Because trials judged to raise some concerns account for a substantial share of the evidence for several outcomes, the certainty of the evidence was downgraded accordingly in the GRADE assessment (48) (Supplementary Table S3).

### 3.4 Meta-analysis results

Nineteen trials were included in the quantitative synthesis; 18 entered the main analysis, and the single trial with an active comparator is described separately. Home-based exercise was compared with an inactive control in 15 trials and with an attention control in three trials. All analyses used a random-effects model with the REML estimator for τ2 and the Hartung–Knapp adjustment, and prediction intervals are reported for outcomes with substantial heterogeneity. The overall results are summarized in Table 3.

#### 3.4.1 Effect of HBE on body mass index and body fat percentage

Four trials (*n* = 197) (35, 54, 58, 63) reported BMI. Heterogeneity was low (I^2^ = 0%), and the pooled mean difference indicated a significant reduction favoring home-based exercise (MD = −0.57 kg/m^2^; 95% CI: −0.71 to −0.43; *p* < 0.001; Figure 3). Because a lower BMI is not universally beneficial, particularly among older women with frailty, this result is reported as a reduction in BMI rather than as an improvement.

**Figure 3:**
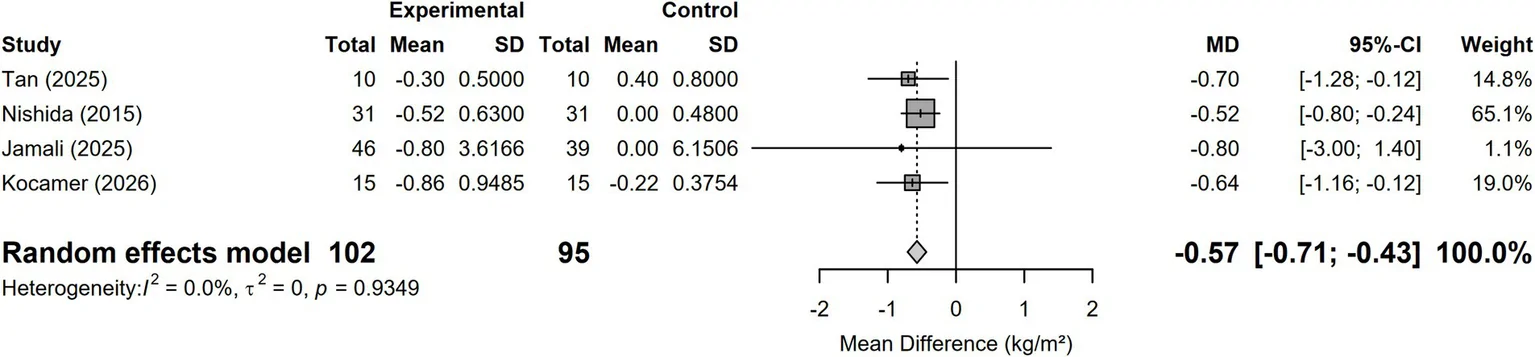
Forest plot of body mass index (BMI). Effect sizes are expressed as mean differences (MDs) in change from baseline with 95% confidence intervals.

Four trials (*n* = 195) (35, 36, 52, 54) reported body fat percentage. The pooled effect did not reach statistical significance (MD = −0.31%; 95% CI: −0.65 to 0.03; I^2^ = 0%; *p* = 0.06; Figure 4). The small number of contributing studies and differences in body composition assessment methods limit interpretation.

**Figure 4:**
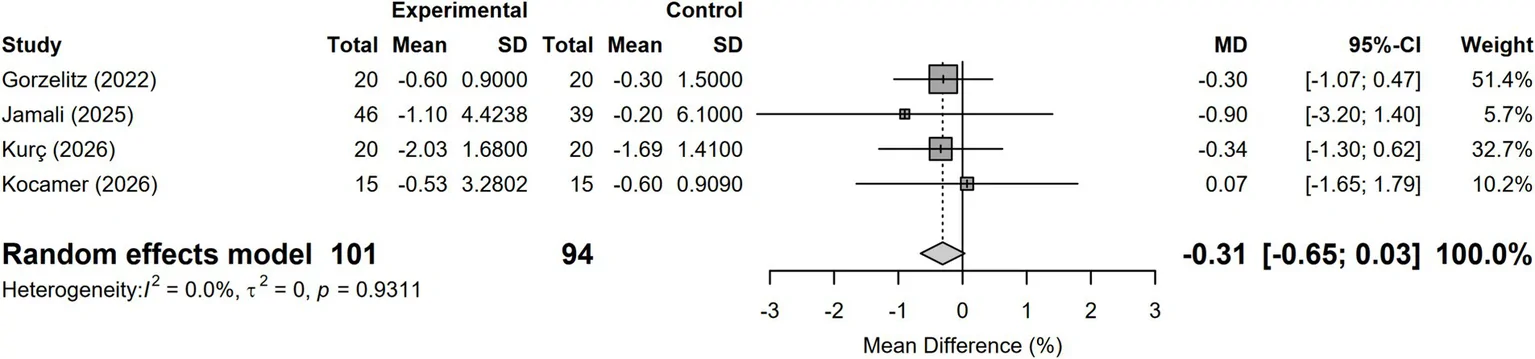
Forest plot of body fat percentage (BFP). Effect sizes are expressed as mean differences (MDs) in change from baseline with 95% confidence intervals.

#### 3.4.2 Effect of HBE on physical function (TUGT, 5 × STS, handgrip strength, and 6MWT)

Four trials (*n* = 261) (42, 51, 61, 62) reported the TUGT. Heterogeneity was substantial (I^2^ = 83%), and the pooled effect did not reach statistical significance (MD = −0.64 s; 95% CI: −1.57 to 0.30; *p* = 0.12; Figure 5). The prediction interval (−2.69 to 1.42 s) crossed the null, indicating that a future trial could plausibly show no effect or a reversal.

**Figure 5:**
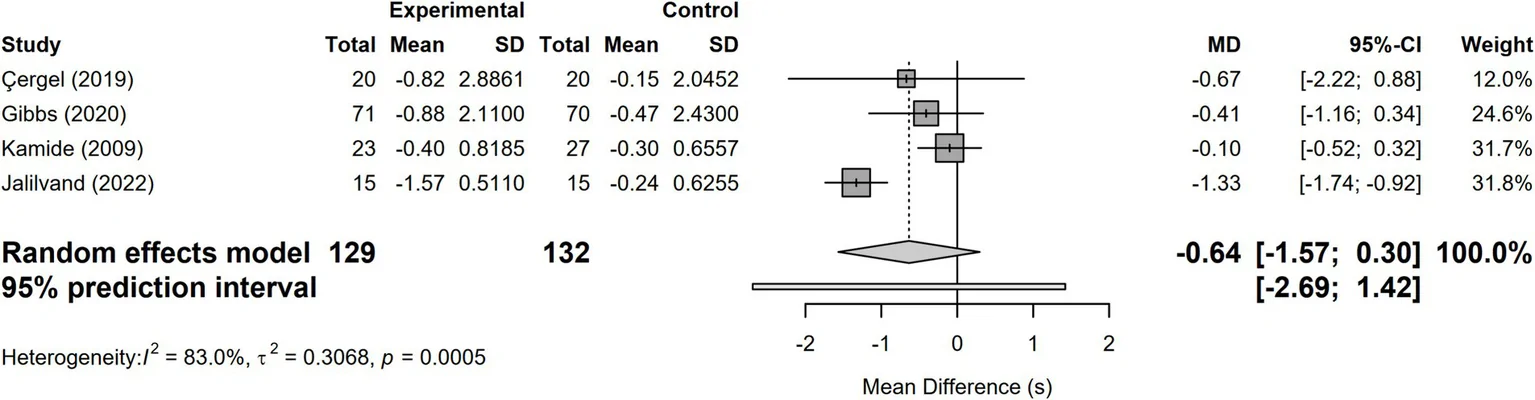
Forest plot of the Timed Up-and-Go Test (TUGT). Effect sizes are expressed as mean differences (MDs) in change from baseline with 95% confidence intervals; the 95% prediction interval is also shown.

Four trials (*n* = 317) (51, 54, 55, 60) reported the 5 × STS. The pooled effect did not reach statistical significance (MD = −1.92 s; 95% CI: −4.36 to 0.52; I^2^ = 43%; *p* = 0.09; Figure 6).

**Figure 6:**
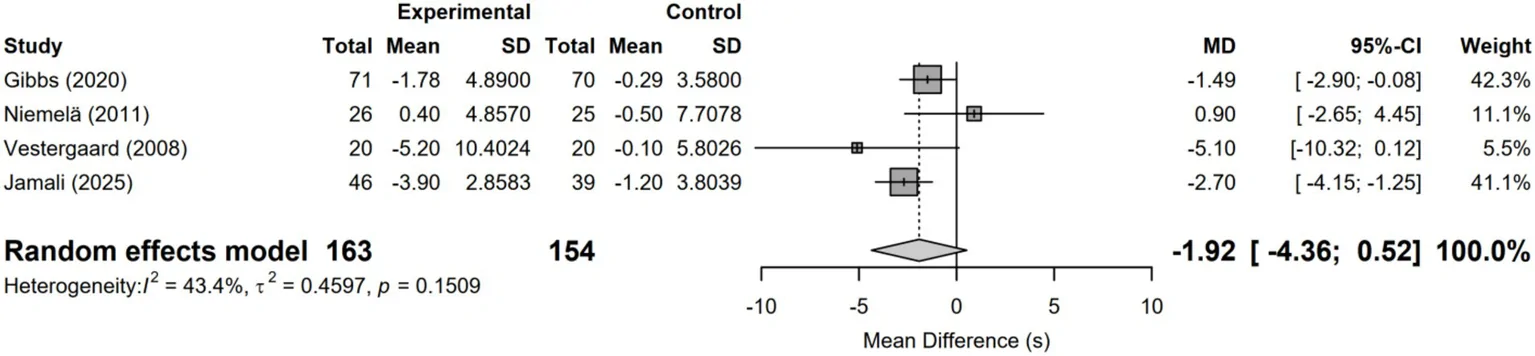
Forest plot of the five-times sit-to-stand test (5 × STS). Effect sizes are expressed as mean differences (MDs) in change from baseline with 95% confidence intervals.

Four trials (*n* = 226) (52, 54, 60, 62) assessed handgrip strength by dynamometry. The pooled effect did not reach statistical significance (MD = 1.43 kg; 95% CI: −1.02 to 3.88; I^2^ = 43%; *p* = 0.16; Figure 7). Handgrip strength should be interpreted as one indicator of muscle function rather than as a comprehensive measure of upper-limb strength.

**Figure 7:**
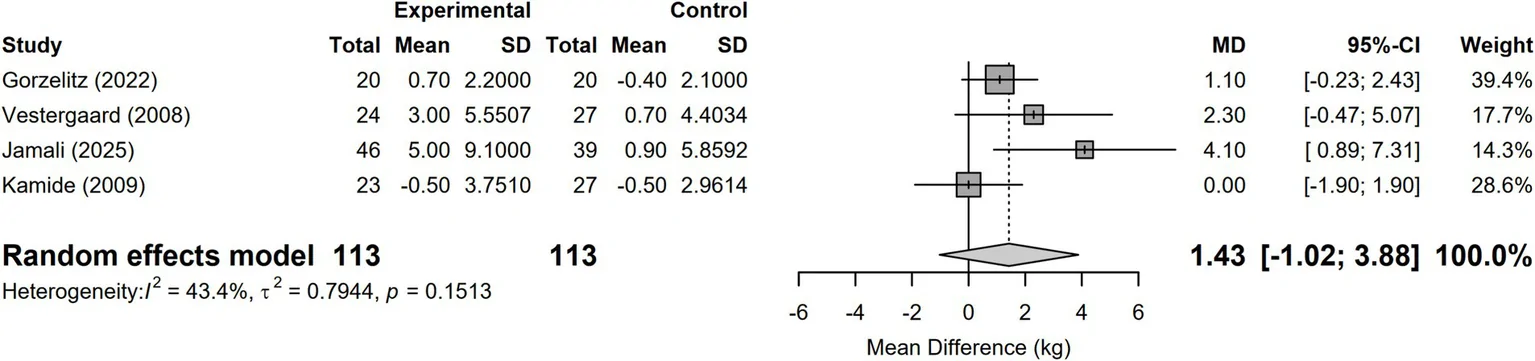
Forest plot of handgrip strength. Effect sizes are expressed as mean differences (MDs) in change from baseline with 95% confidence intervals.

Three trials (*n* = 133) reported the 6MWT (41, 52, 54), and all data were converted to meters. The pooled estimate is reported in Table 3 (MD = 35.16 m; 95% CI: −59.96 to 130.29; Figure 8) together with the individual trial estimates in Supplementary Table S2. It should be interpreted with caution: the contributing trials differed markedly in population and in the scale of change, heterogeneity was substantial (I^2^ = 87%), and the prediction interval (−151.67 to 222.00) crossed the null.

**Figure 8:**
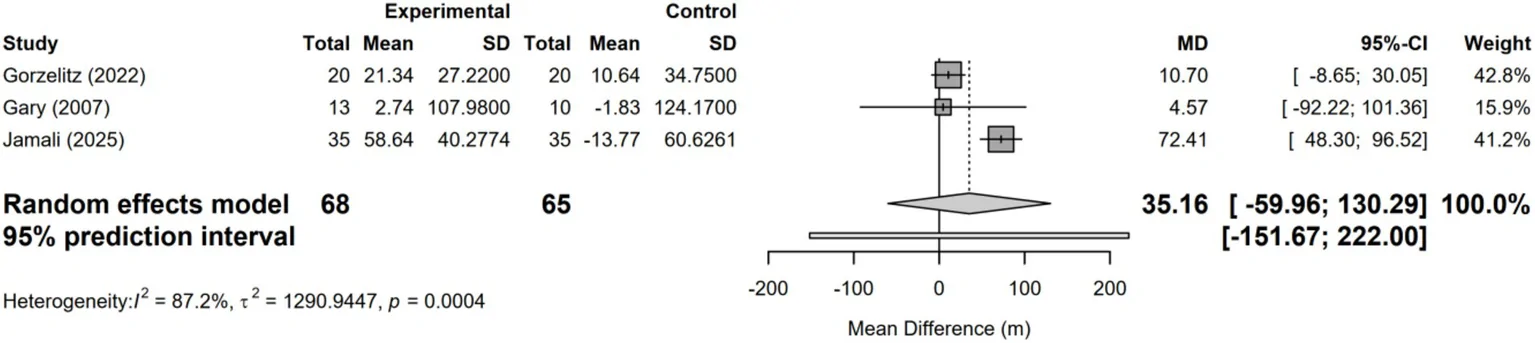
Forest plot of the six-minute walk test (6MWT). Effect sizes are expressed as mean differences (MDs) in change from baseline with 95% confidence intervals; the 95% prediction interval is also shown.

#### 3.4.3 Effect of HBE on cardiometabolic outcomes (triglycerides, SBP, and DBP)

Three trials (*n* = 232) (35, 50, 63) reported serum triglyceride concentrations. The pooled effect did not reach statistical significance (MD = −8.96 mg/dL; 95% CI: −19.45 to 1.52; I^2^ = 0%; *p* = 0.07; Figure 9).

**Figure 9:**
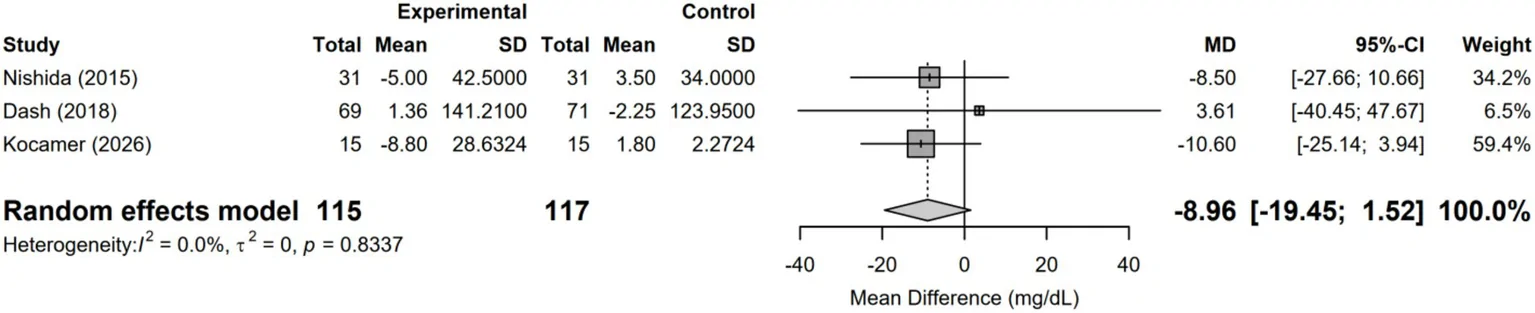
Forest plot of serum triglycerides. Effect sizes are expressed as mean differences (MDs) in change from baseline with 95% confidence intervals.

SBP and DBP were each reported by two trials (54, 59, 63) and were therefore not pooled. The individual estimates are presented in Supplementary Table S2. Neither outcome showed a significant between-group difference, and the confidence intervals were wide.

#### 3.4.4 Effect of HBE on HRQoL

Ten trials (*n* = 463) (35, 36, 41, 42, 49, 52, 53, 56, 57, 60) assessed HRQoL using both generic (SF-36, EQ-5D, FACT-G) and disease-specific (MLHFQ, JOQOL, QUALEFFO-41, FACT-En, EORTC QLQ-C30, MENQOL) instruments; scores were direction-harmonized so that higher values indicate better HRQoL. The pooled effect favored home-based exercise (SMD = 0.51; 95% CI: 0.08 to 0.94; I^2^ = 76%; *p* = 0.025; Figure 10), but the prediction interval (−0.75 to 1.78) crossed the null. In subgroup analysis by instrument type, the effect was 0.35 (95% CI: −0.36 to 1.06) for generic instruments (*k* = 4) and 0.61 (95% CI: −0.13 to 1.35) for disease-specific instruments (*k* = 6), with no significant difference between subgroups (*p* = 0.48; Supplementary Figure 9).

**Figure 10:**
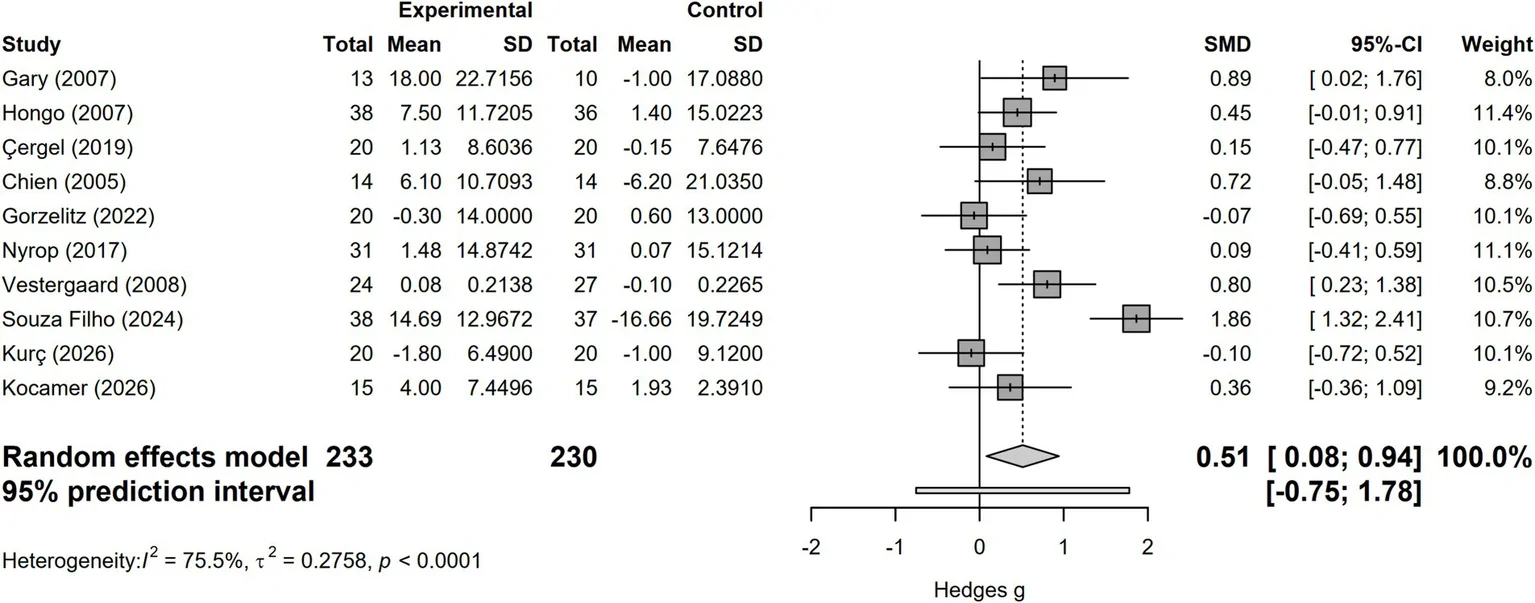
Forest plot of health-related quality of life (HRQoL). Effect sizes are expressed as standardized mean differences (SMDs) in change from baseline with 95% confidence intervals; the 95% prediction interval is also shown.

### 3.5 Subgroup analysis

Exploratory subgroup analyses were performed to examine potential sources of heterogeneity; all strata are reported in Supplementary Table S4, and the analysis for the HRQoL outcome that was judged interpretable is shown in Supplementary Figure 10. For HRQoL, subgroup analysis by comparator tier showed that the eight trials with inactive controls were considerably more homogeneous (I^2^ = 16%) than the two trials with attention controls (I^2^ = 71%), with pooled estimates of 0.29 (95% CI: 0.02 to 0.57) and 1.44 (95% CI: −4.67 to 7.56) respectively. This indicates that much of the heterogeneity in this outcome arises from the two attention-controlled trials, and is consistent with the sensitivity analysis reported in Section 3.6.

Between-subgroup tests were also computed for instrument type, menopausal status ascertainment, comorbidity, session frequency and intervention duration, but the number of trials per stratum ranged from one to nine and these tests are not adjusted for the small number of trials; none of them is therefore interpreted, consistent with the guidance that a between-subgroup difference alone does not demonstrate effect modification. For the TUGT (*k* = 4) and the 6MWT (*k* = 3), subgroup analysis was not informative; the heterogeneity of these outcomes is instead reflected in their prediction intervals, which cross the null.

### 3.6 Sensitivity analyses

Because the risk of bias of the contributing trials should inform the interpretation of the pooled estimates, the analyses were also repeated with the two trials judged to be at high risk of bias overall removed (36, 61). The BMI estimate was unchanged (MD = −0.57 kg/m^2^; 95% CI: −0.71 to −0.43), and the HRQoL estimate remained significant and was slightly larger (SMD = 0.58; 95% CI: 0.12 to 1.04; I^2^ = 76%; *p* = 0.019). The TUGT estimate moved toward the null and its heterogeneity resolved (*k* = 3; MD = −0.20 s; 95% CI: −0.71 to 0.32; I^2^ = 0%), indicating that the heterogeneity of this outcome was attributable largely to that trial; body fat percentage was unchanged in direction and remained non-significant (*k* = 3; MD = −0.29%; 95% CI: −0.98 to 0.39). Restriction of the analyses to the four trials judged to be at low risk of bias was not estimable for any outcome.

Leave-one-out analyses showed that no single trial altered the pooled BMI estimate (Supplementary Figure 1; the corresponding analyses for the remaining outcomes are presented in Supplementary Figures 2–8). For HRQoL, the pooled estimate remained significant after excluding the two studies that reported only a single SF-36 domain (36, 49) (SMD = 0.57; 95% CI: 0.04 to 1.09; I^2^ = 79%; *p* = 0.038; Supplementary Figure 11) and after restricting the analysis to trials with inactive comparators (*k* = 8) (SMD = 0.29; 95% CI: 0.02 to 0.57; I^2^ = 16%; *p* = 0.040). It remained significant, with a wider confidence interval, after excluding the two trials judged to raise some concerns for outcome measurement (52, 53) (SMD = 0.60; 95% CI: 0.06 to 1.13; I^2^ = 79%; *p* = 0.034), but was no longer significant after restricting to trials with documented menopausal status (grades A and B; *k* = 5) (SMD = 0.31; 95% CI: −0.05 to 0.66; I^2^ = 0%; *p* = 0.072) and after excluding all grade C trials (*k* = 7) (SMD = 0.51; 95% CI: −0.11 to 1.12; *p* = 0.089). The HRQoL finding should therefore be interpreted as preliminary.

### 3.7 Publication bias

Publication bias was assessed for HRQoL, the only outcome approaching the minimum number of studies for a funnel plot. Visual inspection of the funnel plot (Figure 11) did not suggest marked asymmetry and Egger’s regression test was not statistically significant (*p* = 0.97); trim-and-fill added no studies. However, with only ten studies and substantial heterogeneity (I^2^ = 76%), the power of this test is limited and publication bias cannot be excluded. Formal assessment was not possible for BMI, TUGT, 5 × STS, handgrip strength, body fat percentage, triglycerides, 6MWT, SBP or DBP because too few studies were available.

**Figure 11:**
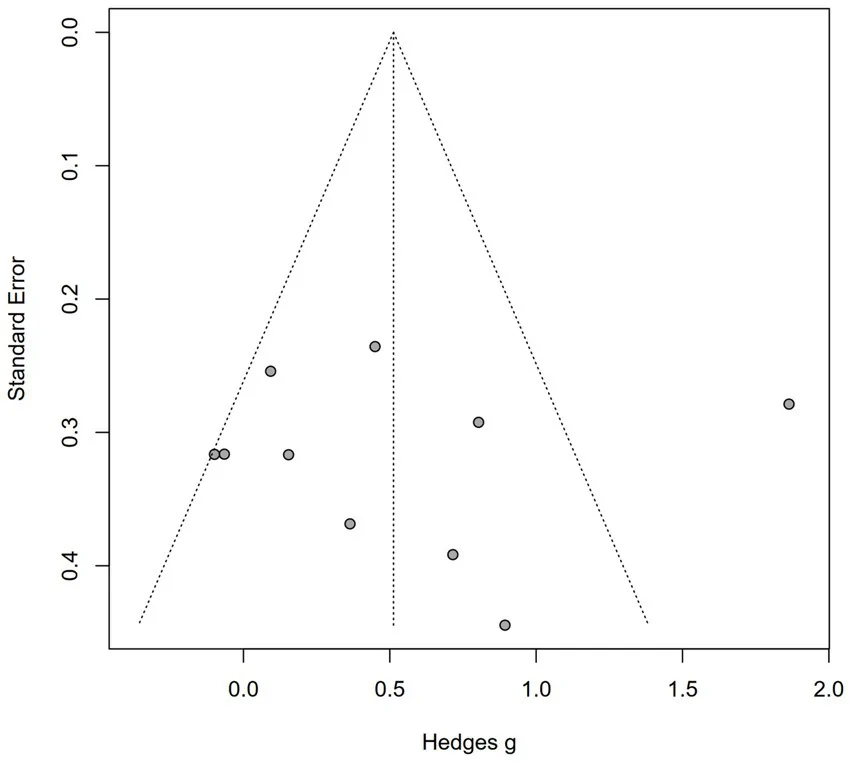
Funnel plot for publication bias assessment of health-related quality of life (HRQoL). The plot was used for visual inspection of publication bias for the HRQoL outcome.

### 3.8 Safety, adherence, and feasibility

Safety, adherence and feasibility were extracted from all included trials and are reported in Table 5. Eight trials explicitly reported no adverse events (35, 36, 49, 56, 58, 60, 62, 65); two reported mild events (54, 57), namely transient generalized soreness, bruising, and myalgia resolving within about 1.5 weeks; and one reported a fall resulting in a forearm fracture together with three cases of back pain during training that resolved on cessation, with no new vertebral fractures (53); the source report did not establish a causal attribution of these events to the exercise program. Eight trials did not report adverse events. Adherence ranged from 66 to 100% where reported; adherence data could not be retrieved in one trial (58) because the application’s usage records were automatically deleted and home exercise logs had not been collected. This review did not directly evaluate feasibility, acceptability or implementation outcomes, and the included trials were not designed to assess them.

## 4 Discussion

### 4.1 Principal findings

This systematic review and meta-analysis included 19 randomized controlled trials involving 1,128 postmenopausal and older women and evaluated the effects of HBE on body composition, physical function, cardiometabolic health, and HRQoL. The pooled results showed that HBE significantly reduced BMI and significantly improved HRQoL. These findings suggest that HBE may reduce body mass index and improve self-reported health status in postmenopausal and older women.

However, the pooled effects of HBE on physical function (TUGT, 5 × STS, handgrip strength and 6MWT) and on cardiometabolic markers (triglycerides and blood pressure) did not reach statistical significance, and the effect on body fat percentage did not reach statistical significance (*p* = 0.06). Overall, the current evidence provides stronger support for a role of HBE in reducing BMI and improving HRQoL than for improving measured physical function or cardiometabolic markers. These findings should be interpreted cautiously because several outcomes were based on a limited number of trials and showed substantial between-study heterogeneity.

### 4.2 Potential mechanisms underlying the effects of HBE

Potential pathways through which HBE could influence functional outcomes include improvements in muscle strength, neuromuscular control, and habitual physical activity. After menopause, declining estrogen levels may impair skeletal muscle protein homeostasis, muscle-bone interactions, and lower-limb strength, thereby contributing to reduced physical function and increased vulnerability to functional decline (6, 9, 12, 66, 67). Even when home-based training is performed at relatively low to moderate intensity, repeated resistance exercises, walking activities, balance tasks, and sit-to-stand movements may provide sufficient stimulus to promote muscular and neuromuscular adaptation.

These adaptations may improve lower-limb strength, postural control, and movement efficiency. Walking-based or aerobic components of HBE may also improve gait economy and cardiorespiratory efficiency, which could contribute to walking capacity, although the pooled effect on the 6MWT did not reach statistical significance in the present analysis. If functional effects are present, they may reflect the combined influence of improved muscle strength, better postural regulation, enhanced walking capacity, and increased engagement in daily physical activity.

### 4.3 Body composition and physical function outcomes

This meta-analysis found that HBE significantly reduced BMI, whereas its effect on BFP was not statistically significant. This finding requires careful interpretation. BMI is an indirect and composite indicator that reflects total body mass relative to height, but it does not distinguish between fat mass, lean mass, and fluid status. If HBE reduces fat mass while maintaining or increasing lean tissue, BMI may decrease without a clear corresponding reduction in BFP. In addition, only four trials reported BFP, the confidence interval was wide and crossed the null, and differences in measurement methods may have reduced statistical power and increased uncertainty.

The magnitude of these effects also needs to be weighed against clinical relevance. The pooled reduction in BMI (0.57 kg/m^2^) is smaller than the minimal important difference of 1.0 kg/m^2^ used in the GRADE assessment, and BMI cannot distinguish a loss of fat mass from a loss of lean tissue. The reductions in body fat percentage (0.31%), TUGT time (0.64 s), handgrip strength (1.43 kg) and triglycerides (8.96 mg/dL) were likewise each smaller than their respective minimal important differences, whereas the reduction in 5 × STS time (1.92 s) exceeded its minimal important difference of 1.5 s but had a confidence interval that crossed the null (Supplementary Table S3). The statistically significant reduction in BMI should therefore not be read as a demonstrated clinical benefit.

Previous evidence suggests that exercise training can improve body composition in postmenopausal women, with aerobic training being more closely associated with fat loss and resistance training contributing to muscle maintenance or gain (16, 21, 22). The present findings are therefore consistent with the view that HBE may support weight-related outcomes, but they also indicate that current evidence is insufficient to confirm a robust effect on BFP.

In terms of physical function, the direction of the pooled estimates was consistent across TUGT, 5 × STS, handgrip strength and 6MWT, although none reached statistical significance and the prediction intervals crossed the null for the most heterogeneous outcomes. TUGT and 5 × STS mainly reflect lower-limb strength, balance control, transfer ability, and functional mobility. Handgrip strength is widely used as an indicator of overall muscle function, whereas 6MWT reflects walking endurance and integrated functional capacity. Taken together, these estimates are too imprecise to establish an effect on measured physical function, and should not be read as evidence of benefit.

### 4.4 Cardiometabolic and HRQoL outcomes

HBE did not show statistically significant effects on triglycerides, SBP, or DBP. Several explanations are possible. First, only a small number of studies contributed data to these outcomes, limiting statistical power. Second, the intensity and duration of many home-based programs may have been insufficient to produce marked cardiometabolic adaptations (68). Third, some participants may have had relatively low baseline cardiometabolic risk, leaving limited room for improvement. Fourth, blood pressure and lipid outcomes may also be influenced by diet, medication use, baseline disease status, and daily lifestyle factors that were not fully controlled in the original trials. This pattern is consistent with a recent meta-analysis of rehabilitation in postmenopausal women, which likewise found no significant pooled effect on blood pressure or on objectively measured physical performance (33). Compared with supervised exercise, HBE often emphasizes safety and long-term adherence, and the actual training intensity may therefore be insufficient to induce strong cardiometabolic adaptation (69, 70).

The pooled effect on HRQoL was statistically significant (SMD = 0.51; 95% CI: 0.08 to 0.94; *p* = 0.025), but the prediction interval crossed the null and the estimate lost significance in two of the prespecified sensitivity analyses (restriction to trials with documented menopausal status and exclusion of all grade C trials). The direction and magnitude are consistent with a recent meta-analysis of exercise and mind–body exercise across the menopausal transition, which reported a large pooled improvement in HRQoL (32). Interpretation is also complicated by differences in clinical background, inconsistency in measurement instruments, and the multidimensional nature of HRQoL. HRQoL is influenced not only by physical function but also by pain, sleep, psychological status, chronic disease burden, and social support. Different instruments may emphasize different domains, thereby increasing between-study heterogeneity. The certainty of this evidence was rated very low on GRADE, and the effect of HBE on HRQoL in postmenopausal and older women should therefore be regarded as uncertain pending further confirmation.

### 4.5 Practical implications

HBE has practical value for postmenopausal and older women who have difficulty participating in facility-based exercise over the long term. For women with travel limitations, financial constraints, limited time, or chronic disease management needs, HBE can serve as an important strategy to promote physical activity. In practical prescription design, aerobic exercise, resistance training, balance exercises and flexibility training may reasonably be combined. It should be noted, however, that the present analysis did not demonstrate statistically significant effects on measured muscle strength, lower-limb function or walking capacity; the recommendation to combine modalities rests on the content of the included programs and on general physical activity guidance rather than on the pooled estimates reported here.

Individualization and safety should be emphasized when HBE is implemented. For women with chronic diseases, osteoporosis, poor physical function, or limited exercise experience, exercise intensity and progression should be adjusted according to health status and baseline fitness. Video instruction, mobile applications, elastic-band training, exercise logs, and home monitoring tools may help improve adherence and training quality. When necessary, remote supervision or periodic evaluation can be used to ensure safety. From this perspective, HBE is not only an alternative exercise format but also a potentially important component of long-term health management and functional maintenance in postmenopausal and older women. For such programs to be reproducible and clinically interpretable, future trials should report exercise prescription using a standardized framework such as the Consensus on Exercise Reporting Template (CERT), so that the delivered dose, progression and supervision can be compared across studies; this issue is discussed further in Section 4.6.

### 4.6 Limitations and future research

This review has several limitations. First, some outcomes were based on a small number of studies, particularly blood pressure, triglycerides, BFP, and 6MWT, which limits the stability of the effect estimates. Second, the included studies differed in exercise modality, training frequency, intervention duration, supervision format, and participant comorbidity profile, which may have contributed to clinical heterogeneity. Third, the original trials provided limited objective reporting of HBE adherence, actual exercise dose, and achieved training intensity, which restricted interpretation of dose–response relationships. Fourth, some studies had incomplete reporting of randomization procedures, inadequate allocation concealment, difficulties with blinding, or potential outcome-measurement bias, which may affect certainty of evidence. Fifth, only English-language studies were included, so language bias cannot be completely excluded. Sixth, because most outcomes included few studies, the ability to assess publication bias was limited. In addition, RoB 2 was applied at the trial level rather than separately for each specific outcome, which may obscure outcome-specific differences in risk of bias, particularly between objective physical-performance outcomes and self-reported HRQoL. Seventh, the registered protocol specified postmenopausal women and excluded women whose menopausal status was not documented, whereas seven trials that enrolled older women without a stated menopause criterion (grade C) were retained, which is a protocol deviation. The search term covering this branch was nested within the menopausal concept and was not developed as a separate search concept, so these trials were not systematically identified and the review should not be read as a complete synthesis of exercise trials in older women irrespective of menopausal status. This deviation has a direct bearing on the results: restricting the analysis to trials with documented menopausal status reduced the HRQoL estimate below statistical significance (SMD = 0.31; 95% CI: −0.05 to 0.66), and the HRQoL finding is reported as preliminary for this reason. Finally, on the GRADE domains of risk of bias, inconsistency, indirectness and imprecision, the certainty of the evidence was moderate for BMI and very low for HRQoL, and low to very low for the remaining outcomes; the conclusions should be read with this in mind.

The difficulty of characterizing the exercise actually delivered is not specific to this review. A recent systematic review of high-intensity resistance and impact training in menopausal women used the Consensus on Exercise Reporting Template (CERT) to appraise 39 trials involving 3,349 women, and reported overall completeness of intervention reporting to be moderate to good (mean 14.3 of 19 items), while items relating to progression, individualization, behavioral support and home-based delivery were frequently omitted (71). Those are precisely the elements on which the present synthesis was least able to draw: progression was not reported, or was explicitly absent, in 6 of the 19 trials; supervision had to be reconstructed from brief descriptions of contact rather than taken from a stated protocol; and adherence was expressed in incompatible ways (session counts, percentages, diary data, or not at all), so that it could not be pooled. The consequence is direct. The inability to characterize the exercise actually delivered is a principal reason why dose–response questions could not be addressed here, and why between-study heterogeneity remained substantial for several outcomes. Routine use of a reporting template such as CERT, alongside explicit specification of what proportion of sessions is delivered at home, would make future syntheses of home-based exercise considerably more informative.

Future research should use larger samples and more rigorous randomized controlled designs to improve evidence quality. Investigators should further standardize HBE prescriptions by clearly reporting exercise type, intensity, frequency, session duration, total intervention duration, and supervision format. Future studies should also strengthen adherence monitoring and adverse-event reporting and should extend follow-up periods to evaluate the durability of intervention effects. For cardiometabolic and HRQoL outcomes, future trials should include adequate sample sizes and use consistent measurement tools where possible. With the development of telemedicine and digital health technology, the roles of video guidance, mobile applications, and wearable devices in HBE management also warrant further investigation.

## 5 Conclusion

Home-based exercise may reduce BMI and improve HRQoL in postmenopausal and older women. The evidence for effects on physical function (TUGT, 5 × STS, handgrip strength, 6MWT) and on cardiometabolic markers (triglycerides, blood pressure) remains inconclusive, with prediction intervals crossing the null for the most heterogeneous outcomes. The certainty of the evidence was moderate for BMI and very low for HRQoL. Before clinical recommendations can be made, further high-quality randomized controlled trials with standardized exercise prescriptions, longer follow-up, and routine reporting of adherence and adverse events are needed.

## Data Availability

The datasets analyzed during this study are included in the article and its Supplementary Material. The data extraction tables and the R script used for all analyses are available from the corresponding author on reasonable request.

## References

[1] World Health Organization. Menopause. Geneva: World Health Organization (2024).

[2] Bień A Niewiadomska I Korżyńska-Piętas M Rzońca E Zarajczyk M Pięta B . General self-efficacy as a moderator between severity of menopausal symptoms and satisfaction with life in menopausal women. Front Public Health. (2024) 12:1426191. doi: 10.3389/fpubh.2024.1426191, 39267631 PMC11390549

[3] Jiang Y Tao M Huang C Lu Q. Menopausal transitional and postmenopausal women’s voices: “what influences their adherence to self-management”: a systematic review and meta-synthesis of qualitative studies. Front Public Health. (2025) 13:1653806. doi: 10.3389/fpubh.2025.1653806, 41235215 PMC12605042

[4] Alnjadat R Momani EA Etoom M Hamdan F ALrub SA. Level of adherence to diet and physical activity among menopausal women and influencing factors in Jordan: a descriptive cross-sectional study. Front Public Health. (2024) 12:1333102. doi: 10.3389/fpubh.2024.1333102 PMC10869606 38371235

[5] Abildgaard J Ploug T Al-Saoudi E Wagner T Thomsen C Ewertsen C . Changes in abdominal subcutaneous adipose tissue phenotype following menopause is associated with increased visceral fat mass. Sci Rep. (2021) 11:14750. doi: 10.1038/s41598-021-94189-2, 34285301 PMC8292317

[6] Geraci A Calvani R Ferri E Marzetti E Arosio B Cesari M. Sarcopenia and menopause: the role of estradiol. Front Endocrinol. (2021) 12:682012. doi: 10.3389/fendo.2021.682012, 34093446 PMC8170301

[7] Ko S-H Jung Y. Energy metabolism changes and dysregulated lipid metabolism in postmenopausal women. Nutrients. (2021) 13:4556. doi: 10.3390/nu13124556, 34960109 PMC8704126

[8] Kozakowski J Gietka-Czernel M Leszczyńska D Majos A. Obesity in menopause — our negligence or an unfortunate inevitability? Prz Menopauzalny. (2017) 16:61–5. doi: 10.5114/pm.2017.68594 PMC5509974 28721132

[9] Lu L Tian L. Postmenopausal osteoporosis coexisting with sarcopenia: the role and mechanisms of estrogen. J Endocrinol. (2023) 259:e230116. doi: 10.1530/JOE-23-0116, 37523234

[10] Opoku AA Abushama M Konje JC. Obesity and menopause. Best Pract Res Clin Obstet Gynaecol. (2023) 88:102348. doi: 10.1016/j.bpobgyn.2023.102348, 37244787

[11] Chang C-F Lee J-I Huang S-P Geng J-H Chen S-C. Regular exercise decreases the risk of osteoporosis in postmenopausal women. Front Public Health. (2022) 10:897363. doi: 10.3389/fpubh.2022.897363, 35784236 PMC9240347

[12] Maltais M Desroches J Dionne IJ. Changes in muscle mass and strength after menopause. J Musculoskelet Neuronal Interact. (2009) 9:186–97. 19949277

[13] González-Gálvez N Moreno-Torres JM Vaquero-Cristóbal R. Resistance training effects on healthy postmenopausal women: a systematic review with meta-analysis. Climacteric. (2024) 27:296–304. doi: 10.1080/13697137.2024.2310521, 38353251

[14] Hsu HH Chiu CY Chen WC Yang YR Wang RY. Effects of exercise on bone density and physical performance in postmenopausal women: a systematic review and meta-analysis. PM R. (2024) 16:1358–83. doi: 10.1002/pmrj.13206, 39032163 PMC11626542

[15] Khalafi M Sakhaei MH Maleki AH Rosenkranz SK Pourvaghar MJ Fang Y . Influence of exercise type and duration on cardiorespiratory fitness and muscular strength in post-menopausal women: a systematic review and meta-analysis. Front Cardiovasc Med. (2023) 10:1190187. doi: 10.3389/fcvm.2023.1190187, 37229231 PMC10204927

[16] Khalafi M Habibi Maleki A Sakhaei MH Rosenkranz SK Pourvaghar MJ Ehsanifar M . The effects of exercise training on body composition in postmenopausal women: a systematic review and meta-analysis. Front Endocrinol. (2023) 14:1183765. doi: 10.3389/fendo.2023.1183765, 37388207 PMC10306117

[17] Mendoza N De Teresa C Cano A Godoy D Hita-Contreras F Lapotka M . Benefits of physical exercise in postmenopausal women. Maturitas. (2016) 93:83–8. doi: 10.1016/j.maturitas.2016.04.017, 27137981

[18] Xu H Liu J Li P Liang Y. Effects of mind-body exercise on perimenopausal and postmenopausal women: a systematic review and meta-analysis. Menopause. (2024) 31:457–67. doi: 10.1097/GME.0000000000002336, 38669625 PMC11465887

[19] Hao H Yuan Y Li J Zhao D Li P Sun J . Association between physical activity and health-related quality of life among adults in China: the moderating role of age. Front Public Health. (2024) 12:1334081. doi: 10.3389/fpubh.2024.1334081, 38601506 PMC11005473

[20] Wang L-Y Chen H-X Zhu H Hu Z-Y Zhou C-F Hu X-Y. Physical activity as a predictor of activities of daily living in older adults: a longitudinal study in China. Front Public Health. (2024) 12:1444119. doi: 10.3389/fpubh.2024.1444119, 39525460 PMC11543459

[21] Bellicha A van Baak MA Battista F Beaulieu K Blundell JE Busetto L . Effect of exercise training on weight loss, body composition changes, and weight maintenance in adults with overweight or obesity: an overview of 12 systematic reviews and 149 studies. Obes Rev. (2021) 22:e13256. doi: 10.1111/obr.13256, 33955140 PMC8365736

[22] Wewege MA Desai I Honey C Coorie B Jones MD Clifford BK . The effect of resistance training in healthy adults on body fat percentage, fat mass and visceral fat: a systematic review and meta-analysis. Sports Med. (2022) 52:287–300. doi: 10.1007/s40279-021-01562-2, 34536199

[23] Bull FC Al-Ansari SS Biddle S Borodulin K Buman MP Cardon G . World Health Organization 2020 guidelines on physical activity and sedentary behavior. Br J Sports Med. (2020) 54:1451–62. doi: 10.1136/bjsports-2020-102955, 33239350 PMC7719906

[24] Mapinduzi J Ndacayisaba G Mitchaï PM Kossi O Bonnechère B. Supervised or home-based? Exploring the best exercise approach for knee osteoarthritis management: a systematic review and meta-analysis. J Clin Med. (2025) 14:525. doi: 10.3390/jcm14020525 PMC11765608 39860530

[25] Solis-Navarro L Gismero A Fernández-Jané C Torres-Castro R Solá-Madurell M Bergé C . Effectiveness of home-based exercise delivered by digital health in older adults: a systematic review and meta-analysis. Age Ageing. (2022) 51:afac243. doi: 10.1093/aging/afac243 PMC9642810 36346736

[26] Mehrabi S Drisdelle S Dutt HR Middleton LE. “If I want to be able to keep going, I must be active.” exploring older adults’ perspectives of remote physical activity supports: a mixed-methods study. Front Public Health. (2024) 12:1328492. doi: 10.3389/fpubh.2024.1328492 PMC10847274 38327585

[27] Peterlin J Dimovski V Colnar S Blažica B Kejžar A. Older adults’ perceptions of online physical exercise management. Front Public Health. (2024) 12:1303113. doi: 10.3389/fpubh.2024.1303113, 38379676 PMC10878307

[28] Toledano-Shubi A Shapira AL Fuchsman R Marco R Hel-Or H Sarig Bahat H. Feasibility and effectiveness of physical exercise for older adults delivered remotely via videoconferencing—systematic review and meta analysis. Age Ageing. (2025) 54:afaf171. doi: 10.1093/aging/afaf171 PMC12206098 40552370

[29] Canton-Martínez E Rentería I Machado-Parra JP Reyes RA Moncada-Jiménez J Johnson DK . A virtually supervised exercise program improved fitness and mental wellness in healthy and comorbidity older adult individuals during the COVID-19 pandemic. Front Public Health. (2024) 12:1328518. doi: 10.3389/fpubh.2024.1328518, 38716241 PMC11074465

[30] Hong J Kim J Kim SW Kong HJ. Effects of home-based tele-exercise on sarcopenia among community-dwelling elderly adults: body composition and functional fitness. Exp Gerontol. (2017) 87:33–9. doi: 10.1016/j.exger.2016.11.002, 27838369

[31] Zhang F Wang Z Su H Zhang X Xu W Li Y. Effect of a home-based resistance exercise program in elderly participants with osteoporosis: a randomized controlled trial. Osteoporos Int. (2022) 33:1937–47. doi: 10.1007/s00198-022-06456-1, 35704055

[32] Ruiz-Cerezo Á Mendoza Laiz N Romero Ferreiro C. Physical exercise and mind-body exercise in the menopausal transition: a systematic review and meta-analysis of effects on quality of life, symptoms, and psychological health. Eur J Obstet Gynecol Reprod Biol. (2026) 325:115338. doi: 10.1016/j.ejogrb.2026.115338, 42520515

[33] Verma A Rishi P Siwach P Yadav B Ray R. Rehabilitation strategies in post menopausal women: a systematic review and meta-analysis of randomized trials. Eur J Obstet Gynecol Reprod Biol. (2026) 325:115278. doi: 10.1016/j.ejogrb.2026.115278, 42401111

[34] Page MJ McKenzie JE Bossuyt PM Boutron I Hoffmann TC Mulrow CD . The PRISMA 2020 statement: an updated guideline for reporting systematic reviews. BMJ. (2021) 372:n71. doi: 10.1136/bmj.n71, 33782057 PMC8005924

[35] Kocamer MY Atılgan E. Effects of supervised reformer pilates and home-based mat pilates on menopausal symptoms and related health outcomes in postmenopausal women with obesity: a randomized controlled trial. Sci Rep. (2026). doi: 10.1038/s41598-026-69442-1

[36] Kurç D Tunalı Van Den Berg AN. Effectiveness of face-to-face and web-based exercise programs on health outcomes in early postmenopausal women: a randomized trial. BMC Womens Health. (2026) 26:297. doi: 10.1186/s12905-026-04457-4, 42035103 PMC13267503

[37] Harlow SD Gass M Hall JE Lobo R Maki P Rebar RW . Executive summary of the stages of reproductive aging workshop +10: addressing the unfinished agenda of staging reproductive aging. Climacteric. (2012) 15:105–14. doi: 10.3109/13697137.2011.650656, 22338612 PMC3580996

[38] Higgins JP Thomas J Chandler J Cumpston M Li T Page MJ . Cochrane Handbook for Systematic Reviews of Interventions. Chichester (UK): John Wiley & Sons (2019). 10.1002/14651858.ED000142 PMC10284251 31643080

[39] Sterne JAC Savović J Page MJ Elbers RG Blencowe NS Boutron I . RoB 2: a revised tool for assessing risk of bias in randomized trials. BMJ. (2019) 366:l4898. doi: 10.1136/bmj.l4898 31462531

[40] Balduzzi S Rücker G Schwarzer G. How to perform a meta-analysis with R: a practical tutorial. Evid Based Ment Health. (2019) 22:153–60. doi: 10.1136/ebmental-2019-300117, 31563865 PMC10231495

[41] Gary R Lee SY. Physical function and quality of life in older women with diastolic heart failure: effects of a progressive walking program on sleep patterns. Prog Cardiovasc Nurs. (2007) 22:72–80. doi: 10.1111/j.0889-7204.2007.05375.x, 17541316

[42] Çergel Y Topuz O Alkan H Sarsan A Sabir Akkoyunlu N. The effects of short-term back extensor strength training in postmenopausal osteoporotic women with vertebral fractures: comparison of supervised and home exercise program. Arch Osteoporos. (2019) 14:82. doi: 10.1007/s11657-019-0632-z, 31352573

[43] Higgins JP Thompson SG Deeks JJ Altman DG. Measuring inconsistency in meta-analyses. BMJ. (2003) 327:557–60. doi: 10.1136/bmj.327.7414.557, 12958120 PMC192859

[44] Hartung J Knapp G. On tests of the overall treatment effect in meta-analysis with normally distributed responses. Stat Med. (2001) 20:1771–82. doi: 10.1002/sim.791, 11406840

[45] IntHout J Ioannidis JPA Rovers MM Goeman JJ. Plea for routinely presenting prediction intervals in meta-analysis. BMJ Open. (2016) 6:e010247. doi: 10.1136/bmjopen-2015-010247, 27406637 PMC4947751

[46] Röver C Knapp G Friede T. Hartung-Knapp-Sidik-Jonkman approach and its modification for random-effects meta-analysis with few studies. BMC Med Res Methodol. (2015) 15:99. doi: 10.1186/s12874-015-0091-1, 26573817 PMC4647507

[47] Sun X Briel M Walter SD Guyatt GH. Is a subgroup effect believable? Updating criteria to evaluate the credibility of subgroup analyses. BMJ. (2010) 340:c117. doi: 10.1136/bmj.c117, 20354011

[48] Guyatt GH Oxman AD Vist GE Kunz R Falck-Ytter Y Alonso-Coello P . GRADE: an emerging consensus on rating quality of evidence and strength of recommendations. BMJ. (2008) 336:924–6. doi: 10.1136/bmj.39489.470347.ad, 18436948 PMC2335261

[49] Chien MY Yang RS Tsauo JY. Home-based trunk-strengthening exercise for osteoporotic and osteopenic postmenopausal women without fracture: a pilot study. Clin Rehabil. (2005) 19:28–36. doi: 10.1191/0269215505cr844oa, 15704506

[50] Dash C Taylor TR Makambi KH Hicks J Hagberg JM Adams-Campbell LL. Effect of exercise on metabolic syndrome in black women by family history and predicted risk of breast cancer: the FIERCE study. Cancer. (2018) 124:3355–63. doi: 10.1002/cncr.31569, 29975403 PMC6108932

[51] Gibbs JC McArthur C Wark JD Thabane L Scherer SC Prasad S . The effects of home exercise in older women with vertebral fractures: a pilot randomized controlled trial. Phys Ther. (2020) 100:662–76. doi: 10.1093/ptj/pzz188, 31899499 PMC7439228

[52] Gorzelitz JS Stoller S Costanzo E Gangnon R Koltyn K Trentham Dietz A . Improvements in strength and agility measures of functional fitness following a telehealth-delivered home-based exercise intervention in endometrial cancer survivors. Support Care Cancer. (2022) 30:447–55. doi: 10.1007/s00520-021-06415-2, 34304292 PMC9362897

[53] Hongo M Itoi E Sinaki M Miyakoshi N Shimada Y Maekawa S . Effect of low-intensity back exercise on quality of life and back extensor strength in patients with osteoporosis: a randomized controlled trial. Osteoporos Int. (2007) 18:1389–95. doi: 10.1007/s00198-007-0398-9, 17572835

[54] Jamali A Molanouri Shamsi M Behmanesh M Kouhkan A Hassani-Abharian P Pourmohammad M . Impact of home-based multi-task exercise training on executive function and TNF/IL-10 ratio in postmenopausal women with diabetes. Cytokine. (2025) 194:157001. doi: 10.1016/j.cyto.2025.157001, 40706194

[55] Niemelä K Väänänen I Leinonen R Laukkanen P. Benefits of home-based rocking-chair exercise for physical performance in community-dwelling elderly women: a randomized controlled trial. Aging Clin Exp Res. (2011) 23:279–87. doi: 10.1007/BF03337754, 20802258

[56] Nyrop KA Callahan LF Cleveland RJ Arbeeva LL Hackney BS Muss HB. Randomized controlled trial of a home-based walking program to reduce moderate to severe aromatase inhibitor-associated arthralgia in breast cancer survivors. Oncologist. (2017) 22:1238–49. doi: 10.1634/theoncologist.2017-0174, 28698390 PMC5634775

[57] Souza Filho BAB Tritany ÉF Matias GHL Smethurst WS Silva Júnior JR Alves JGB. Efficacy of home-based physical exercise on the quality of life of older women with breast cancer in hormoniotherapy: randomized clinical trial. Estud Interdiscip Envelhec. (2024) 29:1–16. doi: 10.22456/2316-2171.135856

[58] Tan A Dunseath G Thomas RL Prior SL Bracken RM Churm R. Effect of home-based exercise with or without a Mediterranean-style diet on adiposity markers in postmenopausal women: a randomized-control trial. Physiol Rep. (2025) 13:e70239. doi: 10.14814/phy2.70239, 39921234 PMC11805804

[59] Vahed A Molanouri Shamsi M Siadat SD Behmanesh M Negaresh R Agh-Mohammadi S . Effects of home-based exercise training on gut microbiota and possible relations with cognitive function and metabolic health in postmenopausal women with type 2 diabetes mellitus: a randomized control trial. Diabetes Res Clin Pract. (2025) 229:112908. doi: 10.1016/j.diabres.2025.112908, 41109629

[60] Vestergaard S Kronborg C Puggaard L. Home-based video exercise intervention for community-dwelling frail older women: a randomized controlled trial. Aging Clin Exp Res. (2008) 20:479–86. doi: 10.1007/BF03325155, 19039291

[61] Ahmadi M Jalilvand M. Effect of an 8-week internet-based aerobic exercise on the balance of older women during the COVID-19 pandemic. Sci J Rehabil Med. (2022) 11:52–63. doi: 10.32598/SJRM.11.1.5

[62] Kamide N Shiba Y Shibata H. Effects on balance, falls, and bone mineral density of a home-based exercise program without home visits in community-dwelling elderly women: a randomized controlled trial. J Physiol Anthropol. (2009) 28:115–22. doi: 10.2114/jpa2.28.115, 19483372

[63] Nishida Y Tanaka K Hara M Hirao N Tanaka H Tobina T . Effects of home-based bench step exercise on inflammatory cytokines and lipid profiles in elderly Japanese females: a randomized controlled trial. Arch Gerontol Geriatr. (2015) 61:443–51. doi: 10.1016/j.archger.2015.06.017, 26228714

[64] Ohta M Hirao N Mori Y Takigami C Eguchi M Tanaka H . Effects of bench step exercise on arterial stiffness in post-menopausal women: contribution of IGF-1 bioactivity and nitric oxide production. Growth Hormon IGF Res. (2012) 22:36–41. doi: 10.1016/j.ghir.2011.12.004, 22245162

[65] Solak S Yeşil H Dündar Ü Toktaş H Yeşil M Korkmaz M. Evaluation of balance exercises on balance, fall risk, and quality of life in postmenopausal women. Turk J Osteoporos. (2022) 28:32–40. doi: 10.4274/tod.galenos.2021.85866

[66] Messier V Rabasa-Lhoret R Barbat-Artigas S Elisha B Karelis AD Aubertin-Leheudre M. Menopause and sarcopenia: a potential role for sex hormones. Maturitas. (2011) 68:331–6. doi: 10.1016/j.maturitas.2011.01.014, 21353405

[67] Beckwée D Delaere A Aelbrecht S Baert V Beaudart C Bruyere O . Exercise interventions for the prevention and treatment of sarcopenia. A systematic umbrella review. J Nutr Health Aging. (2019) 23:494–502. doi: 10.1007/s12603-019-1196-8, 31233069 PMC12280365

[68] Batacan RB Duncan MJ Dalbo VJ Tucker PS Fenning AS. Effects of high-intensity interval training on cardiometabolic health: a systematic review and meta-analysis of intervention studies. Br J Sports Med. (2017) 51:494–503. doi: 10.1136/bjsports-2015-095841, 27797726

[69] Edwards JJ Deenmamode AHP Griffiths M Arnold O Cooper NJ Wiles JD . Exercise training and resting blood pressure: a large-scale pairwise and network meta-analysis of randomized controlled trials. Br J Sports Med. (2023) 57:1317–26. doi: 10.1136/bjsports-2022-106503, 37491419

[70] Wewege MA Thom JM Rye KA Parmenter BJ. Aerobic, resistance or combined training: a systematic review and meta-analysis of exercise to reduce cardiovascular risk in adults with metabolic syndrome. Atherosclerosis. (2018) 274:162–71. doi: 10.1016/j.atherosclerosis.2018.05.002, 29783064

[71] Tortoli E Riccio G Mattii E Ghezzi S Giovannico G Cioeta M. High-intensity resistance and impact exercise in menopausal women: a systematic review of intervention reporting quality and training content. Osteoporos Int. (2026) 37:1945–78. doi: 10.1007/s00198-026-08078-3, 42154220

