# Supplementary material for: Home-based exercise in postmenopausal and older women: effects on body composition, physical function, cardiometabolic health, and quality of life—a systematic review and meta-analysis
Source: Front Public Health. 2026 Oct 29;14:1925532. doi: 10.3389/fpubh.2026.1925532 (PMC13754710; doi:10.3389/fpubh.2026.1925532)
Supplement: Supplementary file 1 [file Table_1.DOCX]

**Supplementary Material**

**Supplementary Table S1. Complete search strategies for the databases.**

| Database | Complete Search Strategy |
| --- | --- |
| PubMed | (((("Exercise"[Mesh]) OR (((((((((((((((((((((((((((Exercises[Title/Abstract]) OR (Exercise, Physical[Title/Abstract])) OR (Exercises, Physical[Title/Abstract])) OR (Physical Exercise[Title/Abstract])) OR (Physical Exercises[Title/Abstract])) OR (Exercise, Isometric[Title/Abstract])) OR (Exercises, Isometric[Title/Abstract])) OR (Isometric Exercises[Title/Abstract])) OR (Isometric Exercise[Title/Abstract])) OR (Exercise, Aerobic[Title/Abstract])) OR (Aerobic Exercise[Title/Abstract])) OR (Aerobic Exercises[Title/Abstract])) OR (Exercises, Aerobic[Title/Abstract])) OR (Exercise Training[Title/Abstract])) OR (Exercise Trainings[Title/Abstract])) OR (Training, Exercise[Title/Abstract])) OR (Trainings, Exercise[Title/Abstract])) OR (Physical Activity[Title/Abstract])) OR (Activities, Physical[Title/Abstract])) OR (Activity, Physical[Title/Abstract])) OR (Physical Activities[Title/Abstract])) OR (Active Breaks[Title/Abstract])) OR (Activity Breaks[Title/Abstract])) OR (Acute Exercise[Title/Abstract])) OR (Acute Exercises[Title/Abstract])) OR (Exercise, Acute[Title/Abstract])) OR (Exercises, Acute[Title/Abstract])))  AND (((home-based[Title/Abstract]) OR (in-home[Title/Abstract])) OR (home[Title/Abstract]))  AND (("Postmenopause"[Mesh]) OR ((((((((((Post-menopausal Period[Title/Abstract]) OR (Period, Post-menopausal[Title/Abstract])) OR (Post menopausal Period[Title/Abstract])) OR (Post-Menopause[Title/Abstract])) OR (Post Menopause[Title/Abstract])) OR (Post-Menopauses[Title/Abstract])) OR (Postmenopausal Period[Title/Abstract])) OR (Period, Postmenopausal[Title/Abstract])) OR (older women[Title/Abstract])) OR (menopausal women[Title/Abstract]))))  AND ((((((randomized controlled trial[Title/Abstract]) OR (randomized[Title/Abstract])) OR (randomly assigned[Title/Abstract])) OR (RCT[Title/Abstract])) OR (controlled trial[Title/Abstract])) OR (clinical trial[Title/Abstract])) |
| Cochrane Library | #1 MeSH descriptor: [Postmenopause] explode all trees  #2 (Post-menopausal Period):ti,ab,kw OR (Period, Post-menopausal):ti,ab,kw OR (Post menopausal Period):ti,ab,kw OR (Post-Menopause):ti,ab,kw OR (Post Menopause):ti,ab,kw OR (Post-Menopauses):ti,ab,kw OR (Postmenopausal Period):ti,ab,kw OR (Period, Postmenopausal):ti,ab,kw OR (older women):ti,ab,kw OR (menopausal women):ti,ab,kw  #3 (home-based):ti,ab,kw OR (in-home):ti,ab,kw OR (home):ti,ab,kw  #4 MeSH descriptor: [Exercise] explode all trees  #5 (Exercises):ti,ab,kw OR (Exercise, Physical):ti,ab,kw OR (Exercises, Physical):ti,ab,kw OR (Physical Exercise):ti,ab,kw OR (Physical Exercises):ti,ab,kw OR (Exercise, Isometric):ti,ab,kw OR (Exercises, Isometric):ti,ab,kw OR (Isometric Exercises):ti,ab,kw OR (Isometric Exercise):ti,ab,kw OR (Exercise, Aerobic):ti,ab,kw OR (Aerobic Exercise):ti,ab,kw OR (Aerobic Exercises):ti,ab,kw OR (Exercises, Aerobic):ti,ab,kw OR (Exercise Training):ti,ab,kw OR (Exercise Trainings):ti,ab,kw OR (Training, Exercise):ti,ab,kw OR (Trainings, Exercise):ti,ab,kw OR (Physical Activity):ti,ab,kw OR (Activities, Physical):ti,ab,kw OR (Activity, Physical):ti,ab,kw OR (Physical Activities):ti,ab,kw OR (Active Breaks):ti,ab,kw OR (Activity Breaks):ti,ab,kw OR (Acute Exercises):ti,ab,kw OR (Acute Exercise):ti,ab,kw OR (Exercise, Acute):ti,ab,kw OR (Exercise, Acute):ti,ab,kw  #6 #1 OR #2  #7 #4 OR #5  #8 #6 AND #7 AND #3 |
| Embase | #1 'postmenopause'/exp  #2 'post-menopausal period':ab,ti OR 'period, post-menopausal':ab,ti OR 'post menopausal period':ab,ti OR 'post-menopause':ab,ti OR 'post menopause':ab,ti OR 'postmenopausal period':ab,ti OR 'post-menopauses':ab,ti OR 'period, postmenopausal':ab,ti OR 'older women':ab,ti OR 'menopausal women':ab,ti  #3 'home-based':ab,ti OR 'in-home':ab,ti OR 'home':ab,ti  #4 #1 OR #2  #5 'exercise'/exp  #6 'exercise, physical':ab,ti OR 'exercises, physical':ab,ti OR 'physical exercise':ab,ti OR 'physical exercises':ab,ti OR 'exercise, isometric':ab,ti OR 'exercises, isometric':ab,ti OR 'isometric exercises':ab,ti OR 'isometric exercise':ab,ti OR 'exercise, aerobic':ab,ti OR 'aerobic exercises':ab,ti OR 'aerobic exercise':ab,ti OR 'exercises, aerobic':ab,ti OR 'exercise training':ab,ti OR 'exercise trainings':ab,ti OR 'training, exercise':ab,ti OR 'trainings, exercise':ab,ti OR 'physical activity':ab,ti OR 'activities, physical':ab,ti OR 'activity, physical':ab,ti OR 'physical activities':ab,ti OR 'active breaks':ab,ti OR 'activity breaks':ab,ti OR 'acute exercise':ab,ti OR 'acute exercises':ab,ti OR 'exercise, acute':ab,ti  #7 #5 OR #6  #8 #3 AND #4 AND #7 |
| Web of Science | #1 ((((((((((TS=(Postmenopause)) OR TS=(Post-menopausal Period)) OR TS=(Period, Post-menopausal)) OR TS=(Post menopausal Period)) OR TS=(Post-Menopause)) OR TS=(Post Menopause)) OR TS=(Post-Menopauses)) OR TS=(Postmenopausal Period)) OR TS=(Period, Postmenopausal)) OR TS=(older women)) OR TS=(menopausal women)  #2 ((TS=(home-based)) OR TS=(in-home)) OR TS=(home)  #3 (((((((((((((((((((((((((((TS=(Exercises)) OR TS=(Exercise, Physical)) OR TS=(Exercises, Physical)) OR TS=(Physical Exercise)) OR TS=(Physical Exercises)) OR TS=(Exercise, Isometric)) OR TS=(Exercises, Isometric)) OR TS=(Isometric Exercises)) OR TS=(Isometric Exercise)) OR TS=(Exercise, Aerobic)) OR TS=(Aerobic Exercise)) OR TS=(Aerobic Exercises)) OR TS=(Exercises, Aerobic)) OR TS=(Exercise Training)) OR TS=(Exercise Trainings)) OR TS=(Training, Exercise)) OR TS=(Trainings, Exercise)) OR TS=(Physical Activity)) OR TS=(Activities, Physical)) OR TS=(Activity, Physical)) OR TS=(Physical Activities)) OR TS=(Active Breaks)) OR TS=(Activity Breaks)) OR TS=(Acute Exercise)) OR TS=(Acute Exercises)) OR TS=(Exercise, Acute)) OR TS=(Exercises, Acute)  #4 ((((TS=(randomized controlled trial)) OR TS=(randomized)) OR TS=(randomly assigned)) OR TS=(controlled trial)) OR TS=(clinical trial)  #5 #1 AND #2 AND #3 AND #4 |

**Supplementary Note. Deviations from the registered protocol**

This review was registered with the International Platform of Registered Systematic Review and Meta-analysis Protocols (INPLASY; INPLASY202640094) on 26 April 2026, by which time the literature search and the quantitative synthesis were substantially complete; the registration is therefore retrospective. The following deviations from the registered protocol are reported.

1. Population. The protocol specified postmenopausal women and stated that women whose menopausal status was not documented would be excluded. In the review as conducted, the population was defined as postmenopausal and older women, and trials that enrolled older women without a documented menopause criterion were retained (classified as grade C in Table 2a). Sensitivity analyses restricted to trials with documented menopausal status (grades A and B) and excluding all grade C trials were added (Section 3.6). The term “older women” was included as a free-text term in the menopausal concept of all four database strategies, but was not developed as a separate older-adult concept, so trials of older women indexed only under other terms would not have been retrieved; the grade C trials are therefore reported as a post hoc extension of the registered population, and the review makes no claim to be a complete synthesis of exercise trials in older women irrespective of menopausal status. The title of the review was changed from “Home-Based Exercise in Postmenopausal Women” to “Home-based exercise in postmenopausal and older women: effects on body composition, physical function, cardiometabolic health, and quality of life—a systematic review and meta-analysis” to reflect the wider population.

**2. Comparator.** The protocol stated that trials with active comparators would be retained. This was implemented, but the single active-comparator trial was not entered into any pooled analysis; it is described separately (Section 3.4).

**3. Choice of model.** The protocol specified that a random-effects model would be applied when I² ≥ 50% and a fixed-effect model otherwise. Because the choice of model should not be driven by the observed heterogeneity, a random-effects model was used for all outcomes irrespective of I² (Section 2.6).

**4. Small-sample adjustment and prediction intervals.** The protocol did not specify a small-sample adjustment. The Hartung–Knapp adjustment, restricted maximum-likelihood estimation of τ² with Q-Profile confidence intervals, and prediction intervals for outcomes with substantial heterogeneity were added (Section 2.6).

**5. Effect measure for the 6MWT.** The protocol specified standardized mean differences for outcomes reported on different scales, including the six-minute walk test. Because the six-minute walk test is measured in units of distance that are directly convertible, all values were converted to meters and pooled as mean differences (Sections 2.6 and 3.4.2).

**6. Subgroup variables.** The protocol pre-specified subgroups by comorbidity, intervention duration and training frequency. Subgroups by comparator tier and by menopausal status ascertainment were added; all subgroup findings are reported as hypothesis-generating only (Section 3.5; Supplementary Table S4).

**7. Additional outcomes reported.** Adherence and adverse events were extracted for all included trials and are summarized in Section 3.8 and Table 2c; the protocol committed only to recording adverse events narratively when reported.

All other elements of the protocol — the remaining eligibility criteria, information sources, search strategy, two-reviewer screening and extraction, the RoB 2 assessment, leave-one-out sensitivity analysis, assessment of reporting bias for outcomes with at least nine trials, and the English-language restriction — were implemented as registered.

**Supplementary Table S2.**

**Individual study estimates for sparsely reported outcomes.**

| Outcome | Study | n (intervention) | n (control) | MD | 95% CI | Unit |
| --- | --- | --- | --- | --- | --- | --- |
| 6MWT | Gorzelitz et al. (2022) | 20 | 20 | 10.7 | −8.65 to 30.05 | meters |
| 6MWT | Gary and Lee (2007) | 13 | 10 | 4.57 | −92.22 to 101.36 | meters |
| 6MWT | Jamali et al. (2025) | 35 | 35 | 72.41 | 48.30 to 96.52 | meters |
| SBP | Nishida et al. (2015) | 31 | 31 | 1.6 | −6.49 to 9.69 | mmHg |
| SBP | Jamali et al. (2025) | 46 | 39 | 0.9 | −6.57 to 8.37 | mmHg |
| DBP | Nishida et al. (2015) | 31 | 31 | -1.5 | −7.94 to 4.94 | mmHg |
| DBP | Jamali et al. (2025) | 46 | 39 | -0.5 | −4.39 to 3.39 | mmHg |

*Note. 6MWT data were converted from feet to meters (×0.3048) and are reported as mean differences in meters.*

*§ The 6MWT change values for this trial are taken from the companion report of the same cohort (Vahed et al. 2025); the change SDs were estimated from pre/post SDs assuming a correlation of r = 0.50, as for the other trials.*

*Each outcome was reported by only two to three trials. SBP and DBP were reported by two trials each and were therefore not pooled; the 6MWT was reported by three trials and is pooled for completeness only. Because the contributing trials differed substantially in population and measurement scale, none of these estimates is interpreted in the text.*

*For transparency, formal pooling of the three 6MWT trials yields MD = 35.16 m (95% CI −59.96 to 130.29, I² = 87%, p = 0.25; prediction interval −151.67 to 222.00); of the two SBP trials, MD = 1.22 mmHg (−3.21 to 5.66, I² = 0%); and of the two DBP trials, MD = −0.77 mmHg (−6.40 to 4.86, I² = 0%).*

*These pooled estimates are based on two to three trials each and are not interpreted in the text.*

**Supplementary Table S3. GRADE evidence profile.**

**GRADE evidence profile — home-based exercise for postmenopausal and older women**

*Prepared from the final analysis (19 trials, 1,128 participants). All data below are the inputs used for the GRADE judgments.*

**Table S3.1. Input data for the GRADE assessment (one row per outcome). Prediction intervals are reported only for outcomes with substantial heterogeneity (I² ≥ 50%); “—” indicates that no prediction interval was calculated.**

| **Outcome** | **k** | **N** | **Effect** | **95% CI** | **Predictioninterval** | **τ²** | **I²** | **Q p** | **p** | **MID** | **Pooled SD** | **OIS** | **Egger p** |
| --- | --- | --- | --- | --- | --- | --- | --- | --- | --- | --- | --- | --- | --- |
| BMI (kg/m²) | 4 | 197 | MD −0.57 | −0.71 to −0.43 | — | 0 | 0% | 0.93 | <0.001 | 1.0 kg/m² | 3.31 | 343 | — |
| Body fat percentage (%) | 4 | 195 | MD −0.31 | −0.65 to 0.03 | — | 0 | 0% | 0.93 | 0.064 | 2.0% | 3.73 | 109 | — |
| HRQoL (SMD) | 10 | 463 | SMD 0.51 | 0.08 to 0.94 | −0.75 to 1.78 | 0.276 | 76% | <0.001 | 0.025 | 0.2 SMD | 1.00 | 784 | 0.97 |
| TUGT (s) | 4 | 261 | MD −0.64 | −1.57 to 0.30 | −2.69 to 1.42 | 0.307 | 83% | <0.001 | 0.118 | 1.0 s | 1.98 | 123 | — |
| 5×STS (s) | 4 | 317 | MD −1.92 | −4.36 to 0.52 | — | 0.460 | 43% | 0.151 | 0.087 | 1.5 s | 5.15 | 369 | — |
| Handgrip strength (kg) | 4 | 226 | MD 1.43 | −1.02 to 3.88 | — | 0.794 | 43% | 0.151 | 0.161 | 2.0 kg | 5.65 | 250 | — |
| Triglycerides (mg/dL) | 3 | 232 | MD −8.96 | −19.45 to 1.52 | — | 0 | 0% | 0.834 | 0.067 | 15 mg/dL | 105.84 | 1561 | — |
| 6MWT (m) | 3 | 133 | MD 35.16 | −59.96 to 130.29 | −151.67 to 222.00 | 1291 | 87% | <0.001 | 0.253 | — | — | — | — |
| SBP (mmHg) | 2 | 147 | MD 1.22 | −3.21 to 5.66 | — | — | 0% | — | — | — | — | — | — |
| DBP (mmHg) | 2 | 147 | MD −0.77 | −6.40 to 4.86 | — | — | 0% | — | — | — | — | — | — |

**Table S3.2. GRADE evidence profile — judgments by domain and overall certainty**

| **Outcome** | **Risk of**  **bias** | **Inconsis-**  **tency** | **Indirect-**  **ness** | **Impre-**  **cision** | **Publication**  **bias** | **Certainty** |
| --- | --- | --- | --- | --- | --- | --- |
| BMI (kg/m²) | serious (−1) | not serious | not serious | not serious | not assessed | MODERATE |
| Body fat percentage (%) | serious (−1) | not serious | not serious | serious (−1) | not assessed | LOW |
| HRQoL (SMD) | serious (−1) | serious (−1) | not serious | serious (−1) | not detected | VERY LOW |
| TUGT (s) | serious (−1) | serious (−1) | not serious | serious (−1) | not assessed | VERY LOW |
| 5×STS (s) | serious (−1) | not serious | not serious | serious (−1) | not assessed | LOW |
| Handgrip strength (kg) | serious (−1) | not serious | not serious | serious (−1) | not assessed | LOW |
| Triglycerides (mg/dL) | serious (−1) | not serious | not serious | very serious (−2) | not assessed | VERY LOW |
| 6MWT (m) | serious (−1) | serious (−1) | not serious | very serious (−2) | not assessed | VERY LOW |
| SBP / DBP (mmHg) | serious (−1) | not serious | not serious | very serious (−2) | not assessed | VERY LOW |

**Table S3.3. Reasons for each judgment**

Risk of bias (all outcomes) — serious (−1). No trial was free of concerns: 4 of the 19 trials were judged at low risk of bias overall (Tan 2025; Çergel 2019; Gibbs et al. 2020; Souza Filho 2024), 13 raised some concerns, and 2 were judged at high risk (Ahmadi and Jalilvand 2022; Kurç et al. 2026). For every outcome, the majority of the trials contributing data raised some concerns or were at high risk. Participants and personnel cannot be blinded in exercise trials, and outcome assessment was unblinded in the majority of trials. The rating is based on the study-level RoB 2 assessment; if risk of bias is assessed per outcome, objective outcomes with blinded assessment may warrant no downgrade.

Inconsistency — serious (−1) for TUGT, HRQoL and 6MWT; not serious elsewhere. TUGT (I² = 83%), HRQoL (I² = 76%) and 6MWT (I² = 87%) showed substantial statistical heterogeneity, and the prediction intervals for TUGT (−2.69 to 1.42 s), HRQoL (−0.75 to 1.78) and 6MWT (−151.67 to 222.00 m) cross the null, meaning that a future trial could plausibly show no effect or a reversal. BMI, body fat percentage and triglycerides showed no heterogeneity (I² = 0%).

Indirectness — not serious. The review question addressed postmenopausal and older women, and 7 of the 19 trials enrolled older women without documenting menopausal status (grade C). These trials therefore provide direct evidence for the review question. If the question were restricted to women with documented menopausal status, indirectness would be rated serious (−1).

**Imprecision — one or two levels depending on the outcome.** Downgraded by one level where the 95% confidence interval crosses the null (TUGT, 5×STS, handgrip strength, body fat percentage) and for HRQoL, where the lower confidence bound (0.08) lies below the MID of 0.2 SMD even though the interval excludes the null. Downgraded by two levels for triglycerides (k = 3, N = 232 against an OIS of 1,561, i.e. < 50% of the OIS) and for 6MWT, SBP and DBP (k = 2–3). No downgrade was applied to BMI: although N = 197 is below the OIS of 343, the confidence interval is narrow and lies wholly below the MID in magnitude (−0.71 to −0.43 kg/m²), so the effect is precisely estimated, though small.

**Publication bias — not detected / not assessed.** Egger's regression test could be performed only for HRQoL (k = 10, the minimum for a funnel plot): p = 0.97, with no studies added by trim-and-fill. The test has limited power at this number of studies and with I² = 76%, so publication bias cannot be excluded. Formal assessment was not possible for any other outcome.

**Notes**

1. All RCTs start at HIGH certainty; each serious limitation subtracts one level and each very serious limitation subtracts two. Four levels are possible: high, moderate, low, very low.
2. All estimates are from random-effects models (REML estimator for τ²) with the Hartung–Knapp small-sample adjustment. Prediction intervals are from the t-distribution with df = k − 1 and were computed for outcomes with substantial heterogeneity.
3. OIS (optimal information size) = 4 × (1.96 + 0.84)² × SD² / MID² for α = 0.05 and power = 80%, where MID is the minimal important difference; for SMD, SD = 1 by definition. The minimal important differences used here are conventional thresholds adopted for this purpose, not values derived from the included trials.
4. The risk-of-bias rating is applied to all outcomes equally because the RoB 2 assessment was performed at study level. This is a limitation of the present review.
5. The two outcomes that reached statistical significance are graded MODERATE (BMI) and VERY LOW (HRQoL). For HRQoL the rating reflects substantial unexplained heterogeneity, a prediction interval crossing the null, and a confidence interval whose lower bound falls below the MID; the effect also does not survive restriction to trials with documented menopausal status.
6. SBP and DBP were each reported by two trials and were therefore not pooled; the 6MWT was reported by three trials and is pooled for completeness. Individual study estimates for all three outcomes are given in Supplementary Table S2, and their certainty ratings are provided for completeness.

**Supplementary Table S4. Exploratory subgroup analyses.**

*All subgroup analyses were exploratory and hypothesis-generating. Between-subgroup differences are reported for completeness and are not interpreted as evidence of effect modification, consistent with the small number of trials per stratum. Analyses were performed with a random-effects model (REML estimator for τ²) with the Hartung–Knapp small-sample adjustment. τ² and I² are not estimable for strata containing a single trial. HRQoL, health-related quality of life; MD, mean difference; SMD, standardized mean difference. IMPORTANT: the between-subgroup tests are not adjusted for the small number of trials and are anti-conservative when strata contain only one to three trials; they are reported for transparency only and must not be interpreted as evidence of effect modification. Where two strata have clearly overlapping confidence intervals, the corresponding between-subgroup p-value should not be relied upon.*

| **Outcome** | **Subgroup variable** | **Level** | **k** | **Estimate (95% CI)** | **τ²** | **I²** | **p (between subgroups)** |
| --- | --- | --- | --- | --- | --- | --- | --- |
| **HRQoL (Hedges g)** | Comorbidity | With comorbidity | 9 | 0.58 (0.12 to 1.04) | 0.2716 | 75.5% | 0.0687 |
| HRQoL (Hedges g) | Comorbidity | Without comorbidity | 1 | -0.10 (-0.72 to 0.52) | — | Not estimable | 0.0687 |
| HRQoL (Hedges g) | Comparator tier | Attention control | 2 | 1.44 (-4.67 to 7.56) | 0.3331 | 70.8% | 0.0205 |
| HRQoL (Hedges g) | Comparator tier | Inactive control | 8 | 0.29 (0.02 to 0.57) | 0.0168 | 15.8% | 0.0205 |
| HRQoL (Hedges g) | Intervention duration | 9–12 weeks | 4 | 0.86 (-0.44 to 2.16) | 0.5795 | 86.0% | 0.0128 |
| HRQoL (Hedges g) | Intervention duration | >12 weeks | 2 | 0.59 (-1.61 to 2.79) | 0 | 0.0% | 0.0128 |
| HRQoL (Hedges g) | Intervention duration | ≤8 weeks | 4 | 0.11 (-0.16 to 0.38) | 0 | 0.0% | 0.0128 |
| HRQoL (Hedges g) | Menopausal status ascertainment | Grade A | 4 | 0.23 (-0.30 to 0.76) | 0 | 0.0% | 0.6914 |
| HRQoL (Hedges g) | Menopausal status ascertainment | Grade B | 1 | 0.45 (-0.01 to 0.91) | — | Not estimable | 0.6914 |
| HRQoL (Hedges g) | Menopausal status ascertainment | Grade B* | 2 | 0.97 (-10.28 to 12.23) | 1.4972 | 95.5% | 0.6914 |
| HRQoL (Hedges g) | Menopausal status ascertainment | Grade C | 3 | 0.52 (-0.81 to 1.84) | 0.1755 | 60.4% | 0.6914 |
| HRQoL (Hedges g) | Session frequency | 21 sessions/week | 1 | 0.72 (-0.05 to 1.48) | — | Not estimable | 0.3847 |
| HRQoL (Hedges g) | Session frequency | 2 sessions/week | 2 | 0.12 (-2.58 to 2.81) | 0 | 0.0% | 0.3847 |
| HRQoL (Hedges g) | Session frequency | 3 sessions/week | 4 | 0.41 (-0.36 to 1.17) | 0.1191 | 51.8% | 0.3847 |
| HRQoL (Hedges g) | Session frequency | 5 sessions/week | 2 | 1.15 (-7.84 to 10.13) | 0.9336 | 93.3% | 0.3847 |
| HRQoL (Hedges g) | Session frequency | Other/not reported | 1 | 0.09 (-0.41 to 0.59) | — | Not estimable | 0.3847 |
| **BMI (MD)** | Comorbidity | With comorbidity | 2 | -0.65 (-1.10 to -0.20) | 0 | 0.0% | 0.2260 |
| BMI (MD) | Comorbidity | Without comorbidity | 2 | -0.55 (-1.44 to 0.34) | 0 | 0.0% | 0.2260 |
| BMI (MD) | Intervention duration | 9–12 weeks | 2 | -0.52 (-0.97 to -0.08) | 0 | 0.0% | 0.0020 |
| BMI (MD) | Intervention duration | ≤8 weeks | 2 | -0.67 (-1.04 to -0.29) | 0 | 0.0% | 0.0020 |
| BMI (MD) | Menopausal status ascertainment | Grade A | 2 | -0.67 (-1.04 to -0.29) | 0 | 0.0% | 0.0020 |
| BMI (MD) | Menopausal status ascertainment | Grade B | 2 | -0.52 (-0.97 to -0.08) | 0 | 0.0% | 0.0020 |
| **Body fat percentage (MD)** | Intervention duration | 9–12 weeks | 2 | -0.36 (-2.65 to 1.93) | 0 | 0.0% | 0.6398 |
| Body fat percentage (MD) | Intervention duration | ≤8 weeks | 2 | -0.24 (-2.46 to 1.97) | 0 | 0.0% | 0.6398 |
| **TUGT (MD)** | Comorbidity | With comorbidity | 2 | -0.46 (-1.76 to 0.84) | 0 | 0.0% | 0.6812 |
| TUGT (MD) | Comorbidity | Without comorbidity | 2 | -0.72 (-8.53 to 7.10) | 0.7122 | 94.1% | 0.6812 |
| TUGT (MD) | Comparator tier | Attention control | 1 | -0.41 (-1.16 to 0.34) | — | Not estimable | 0.5879 |
| TUGT (MD) | Comparator tier | Inactive control | 3 | -0.71 (-2.40 to 0.98) | 0.4420 | 88.3% | 0.5879 |
| TUGT (MD) | Intervention duration | >12 weeks | 2 | -0.17 (-1.84 to 1.50) | 0 | 0.0% | < 0.0001 |
| TUGT (MD) | Intervention duration | ≤8 weeks | 2 | -1.29 (-3.35 to 0.78) | 0 | 0.0% | < 0.0001 |
| TUGT (MD) | Menopausal status ascertainment | Grade A | 1 | -0.67 (-2.22 to 0.88) | — | Not estimable | 0.9619 |
| TUGT (MD) | Menopausal status ascertainment | Grade C | 3 | -0.63 (-2.26 to 1.01) | 0.3870 | 88.7% | 0.9619 |
| **5×STS (MD)** | Comparator tier | Attention control | 1 | -1.49 (-2.90 to -0.08) | — | Not estimable | 0.7264 |
| 5×STS (MD) | Comparator tier | Inactive control | 3 | -2.08 (-8.61 to 4.45) | 3.4004 | 55.6% | 0.7264 |
| 5×STS (MD) | Menopausal status ascertainment | Grade B | 1 | -2.70 (-4.15 to -1.25) | — | Not estimable | 0.3122 |
| 5×STS (MD) | Menopausal status ascertainment | Grade C | 3 | -1.38 (-6.02 to 3.27) | 0.5020 | 43.8% | 0.3122 |
| **Handgrip strength (MD)** | Comorbidity | With comorbidity | 3 | 2.01 (-1.54 to 5.57) | 0.8679 | 35.6% | 0.1133 |
| Handgrip strength (MD) | Comorbidity | Without comorbidity | 1 | 0.00 (-1.90 to 1.90) | — | Not estimable | 0.1133 |
| Handgrip strength (MD) | Intervention duration | 9–12 weeks | 2 | 2.23 (-16.24 to 20.70) | 2.9285 | 65.1% | 0.4756 |
| Handgrip strength (MD) | Intervention duration | >12 weeks | 2 | 0.92 (-13.39 to 15.23) | 1.1758 | 44.5% | 0.4756 |
| Handgrip strength (MD) | Menopausal status ascertainment | Grade B | 1 | 4.10 (0.89 to 7.31) | — | Not estimable | 0.0658 |
| Handgrip strength (MD) | Menopausal status ascertainment | Grade C | 3 | 0.95 (-1.24 to 3.13) | <0.0001 | 0.0% | 0.0658 |

**Supplementary Figures**

Supplementary Figure 1. Leave-one-out sensitivity analysis for body mass index (BMI). Effect sizes are presented as mean differences (MDs) in change from baseline with 95% confidence intervals.

Supplementary Figure 2. Leave-one-out sensitivity analysis for the Timed Up-and-Go test (TUGT). Effect sizes are presented as mean differences (MDs) in change from baseline with 95% confidence intervals.

Supplementary Figure 3. Leave-one-out sensitivity analysis for the five-times sit-to-stand test (5×STS). Effect sizes are presented as mean differences (MDs) in change from baseline with 95% confidence intervals.

Supplementary Figure 4. Leave-one-out sensitivity analysis for handgrip strength. Effect sizes are presented as mean differences (MDs) in change from baseline with 95% confidence intervals.

Supplementary Figure 5. Leave-one-out sensitivity analysis for health-related quality of life (HRQoL). Effect sizes are presented as standardized mean differences (SMDs) in change from baseline with 95% confidence intervals.

Supplementary Figure 6. Leave-one-out sensitivity analysis for body fat percentage. Effect sizes are presented as mean differences (MDs) in change from baseline with 95% confidence intervals.

Supplementary Figure 7. Leave-one-out sensitivity analysis for serum triglycerides. Effect sizes are presented as mean differences (MDs) in change from baseline with 95% confidence intervals.

Supplementary Figure 8. Leave-one-out sensitivity analysis for six-minute walk test (6MWT) distance. Effect sizes are presented as mean differences (MDs) in change from baseline with 95% confidence intervals.

Supplementary Figure 9. Subgroup analysis of the effect of instrument type on health-related quality of life (HRQoL), comparing generic with disease-specific instruments. Effect sizes are presented as standardized mean differences (SMDs) in change from baseline with 95% confidence intervals.

Supplementary Figure 10. Subgroup analysis of the effect of comparator tier on health-related quality of life (HRQoL), comparing inactive with attention controls. Effect sizes are presented as standardized mean differences (SMDs) in change from baseline with 95% confidence intervals. Arrows indicate confidence intervals extending beyond the plotted range. The between-subgroup difference was not interpreted.

Supplementary Figure 11. Sensitivity analysis for health-related quality of life (HRQoL) after excluding the two trials that reported only a single SF-36 domain (Chien 2005; Kurç 2026). Effect sizes are presented as standardized mean differences (SMDs) in change from baseline with 95% confidence intervals.
